# Biosurveillance of Invasive Southern Corn Rust: Insights Into Recent Migration Patterns and Virulence Variation

**DOI:** 10.1111/mpp.70159

**Published:** 2025-09-30

**Authors:** Yuanjie Li, Wiruda Pootakham, Supawadee Ingsriswang, Fe Dela Cueva, Benjamine William Cordez, Yusufjion Gafforov, Jintana Unartngam, Lin Liu, Guozhi Bi, Peng Zhao, K. M. Tsui Clement, Junmin Liang, Lei Cai

**Affiliations:** ^1^ State Key Laboratory of Microbial Diversity and Innovative Utilization, Institute of Microbiology Chinese Academy of Sciences Beijing China; ^2^ College of Life Sciences University of Chinese Academy of Sciences Beijing China; ^3^ National Science and Technology Development Agency (NSTDA) National Center for the Genetic Engineering and Biotechnology (BIOTEC) Pathum Thani Thailand; ^4^ Institute of Plant Breeding, College of Agriculture and Food Science University of the Philippines Los Baños, College Los Baños Laguna Philippines; ^5^ Central Asian Center for Development Studies New Uzbekistan University Tashkent Uzbekistan; ^6^ Department of Plant Pathology, Faculty of Agriculture at Kamphaeng Saen Kasetsat University Nakhon Pathom Thailand; ^7^ State Key Laboratory for Conservation and Utilization of Bio‐Resources in Yunnan Yunnan Agricultural University Kunming China; ^8^ State Key Laboratory of Plant Environmental Resilience, College of Biological Sciences China Agricultural University Beijing China; ^9^ Division of Infectious Diseases, Faculty of Medicine University of British Columbia Vancouver British Columbia Canada; ^10^ National Centre for Infectious Diseases Tan Tock Seng Hospital Singapore Singapore; ^11^ LKC School of Medicine Nanyang Technological University Singapore Singapore

**Keywords:** effector variation, host selection, population transcriptomics, *Puccinia polysora*, trajectory tracking

## Abstract

Emerging pathogen races spreading via long‐distance migration increasingly threaten global agricultural ecosystems. Understanding how pathogens migrate and adapt to new hosts via virulence evolution is crucial for developing strategies to mitigate future crop damage. Here we performed biosurveillance of *Puccinia polysora*, a global fungal pathogen causing southern corn rust (SCR), across China, Thailand and the Philippines. By analysing 193 field transcriptomic data, we detected both epidemic and endemic lineages co‐circulating in each country and elucidated the crucial role of host selection in driving the diversification of endemic lineages. Gene flow assessments and trajectory tracking indicated that the SCR infection source in northern China is likely of domestic origin and pathogen migration from the Philippines/Thailand into China is restricted to Hainan, coastal Guangdong and southern Yunnan. We detected country‐specific variants in 32 effector genes, with *AvrRppC* exhibiting the strongest positive selection. A phylogenetically distinct Luzon Island lineage (Philippines), carrying a novel *AvrRppC* allele capable of overcoming *RppC*‐mediated resistance and represents a potentially invasive threat. Finally, we reviewed the global migration history of *P. polysora* in light of our findings. Our work represents the first step toward establishing an international surveillance network for *P. polysora* and emphasised a comprehensive control strategy integrating local governance and invasion prevention of international races.

## Graphical abstract

Historical dispersal of *Puccinia polysora* and its phylogenetic differentiation and variation dynamics in the complementary epidemic centre of East and Southeast Asia.

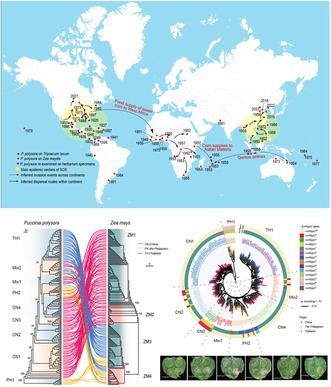

## 1 Introduction

The invasion of plant‐pathogenic fungi represents a significant threat to agricultural and forestry ecosystems worldwide. These invasive pathogens not only jeopardise food security but also drive native plant populations to decline and potential extinction (Gladieux et al. 2015). Understanding the invasion biology of fungal pathogens not only improves the fundamental understanding of evolutionary processes, but also facilitates disease control and pest or disease management (Latorre et al. 2023). Spores of fungal pathogens can be dispersed by various means, including both natural short‐distance spread via rain, water, soil, animals or humans and long‐distance air transmission mechanisms. Atmospheric currents, combined with intensifying human‐mediated activities such as global trade and tourism, have also largely enhanced the potential for transcontinental movement of fungal pathogens (Golan and Pringle 2017). Elucidating the invasion history, source populations and the distribution of genetic variability in invasive species is crucial for predicting their future establishment and adaptive potential (Acevedo‐Limón et al. 2020).

Rust fungi (Pucciniales) have been renowned for their remarkable spore dispersal capacity and ability to trigger devastating epidemics following invasion events (Hovmøller et al. 2023). Historical records documented several high‐impact long‐distance transcontinental dispersal events involving rust pathogens. A particularly alarming case is *Puccinia graminis* f. sp. *tritici* race TTKSK (Ug99), the causal agent of wheat stem rust, which has spread from East Africa to the Middle East since 1999 (Pretorius et al. 2000) and more recently to Nepal in 2024 (Patpour et al. 2024), posing a significant threat to wheat production throughout Europe and Asia. Similarly, the ‘Warrior’ race of wheat stripe rust pathogen (*Puccinia striiformis*) has rapidly expanded across Europe, severely jeopardising local wheat yields (Walter et al. 2016). These increasing transcontinental invasions prompted the establishment of the Borlaug Global Rust Initiative, an international effort to enhance pathogen surveillance and control strategies (McIntosh and Pretorius 2011).

More recently, southern corn rust (SCR, caused by *Puccinia polysora*) has emerged as a growing threat to global maize production and is predicted to have the potential to become one of the most severe maize diseases in the future (Ramirez‐Cabral et al. 2017; Ma et al. 2024). In China, SCR initially exhibited a restricted geographic distribution, being confined to southern tropical regions including Taiwan and Hainan during the 1970s (Duan and He 1984). However, epidemiological surveillance has reported a significant range expansion over the past two decades; multiple epidemics in central China have elevated SCR to a top‐priority maize disease (Li et al. 2025; Liu et al. 2016). A recent population study reported the emergence of a highly virulent lineage (G6) within the Chinese SCR population, which has demonstrated the capacity to overcome multiple major resistance genes, posing new challenges for maize production (Li et al. 2025). The geographic origins of SCR populations in China remain unknown. Wang et al. (2020) speculated a potential Southeast Asian origin but lack empirical support from population genetic or epidemiological data. Addressing these knowledge gaps through comprehensive regional surveillance will provide important information for revealing the historical migration routes of SCR, characterising the current patterns of genetic diversity across populations, as well as identifying potential sources of novel virulence, which is critical for modelling pathogen risk prediction and informing pre‐emptive breeding strategies.

Pathogenomics, which leverages next‐generation sequencing technologies, has emerged as a preferred method for monitoring and detecting genetic variation, particularly in organisms with small genome sizes, such as viruses (avg. ~50 kb), bacteria or filamentous fungi with small genome sizes (< 50 Mb) (Hasman et al. 2014; Gladieux et al. 2018; Shuai et al. 2022). In several rust species, such as *P. striiformis* (Brar et al. 2018), *Puccinia coronata* (Miller et al. 2020) and *P. triticina* (Sperschneider et al. 2023), whole‐genome sequencing has been employed to investigate population divergence and variation. However, these rust species have relatively small genome sizes (~100 Mb for haploid) in Pucciniales (avg. ~380 Mb, the largest known up to 2.4 Gb). As a striking contrast, *P. polysora* possesses a significantly larger genome, reaching up to 1.71 Gb (Liang et al. 2023). Alternatively, RNA sequencing (RNA‐seq) has emerged as a useful complementary tool in population genomic studies. By focusing on the expressed fraction of the genome, this approach offers enhanced cost efficiency compared to whole‐genome sequencing, making it particularly suitable for large‐scale investigations of genetic variation underlying adaptive divergence at both sequence and gene expression levels (Alvarez et al. 2015; West‐Eberhard 2005). Moreover, RNA‐seq of infected leaves yields transcriptome data for both the pathogen and host in situ (Westermann et al. 2012), which is advantageous for studying co‐evolutionary dynamics and interactions between rust pathogens and their hosts.

In this study, we carried out an international surveillance of SCR in China, Thailand and the Philippines. A total of 193 field transcriptomic data were collected to study genetic relationships and variations in virulence profiles and adaptation. Our findings revealed both pandemic and endemic lineages within each national population. Notably, a highly divergent population identified from Luzon Island, Philippines, could potentially circumvent the major maize resistance present in China. Metatranscriptomic data revealed that host selection played a significant role in shaping endemic lineages. To elucidate the patterns of international movement and transmission of SCR, we performed gene flow analyses and trajectory tracking incorporating meteorological data. Collectively, our results indicated limited dissemination of *P. polysora* from Thailand and the Philippines. Furthermore, to lay the foundation for early warning systems targeting pathogen race invasion and succession dynamics, we conducted an extensive analysis of virulence variation. Our results supported the northward spread and expansion from southern (Guangdong/Guangxi) populations through central (Fujian/Jiangxi) to northern regions (Shandong/Henan) in China. The migration network in East Asia and Southeast Asia (one of the current epidemic centres) delineated in this study provides a crucial piece of the puzzle for the global migration map of *P. polysora*.

## 2 Results

### 2.1 Coexistence of Epidemic and Endemic Populations

**FIGURE 1:**
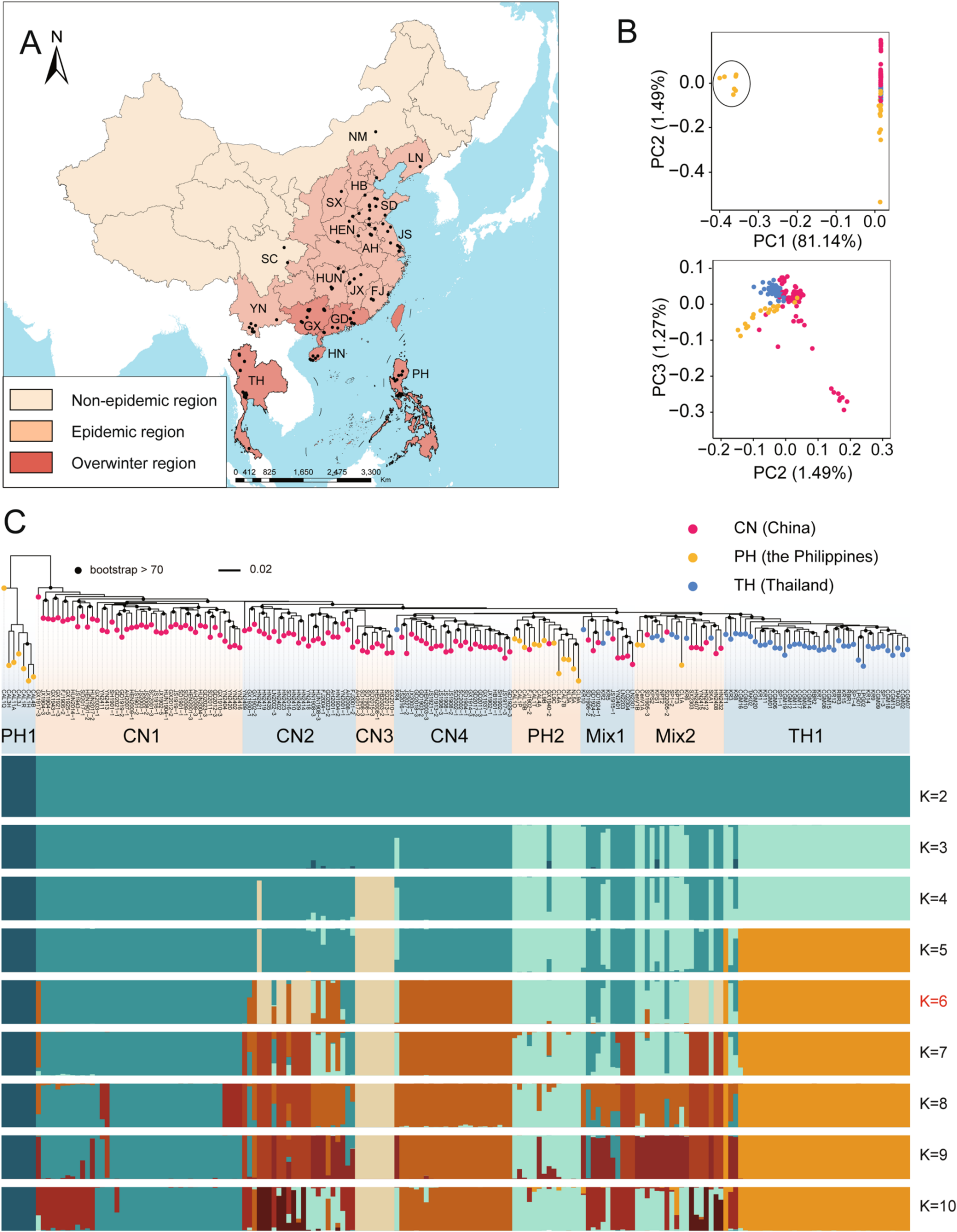
Isolates sampling and population structure of *Puccinia polysora* in this study. (A) Geographic distribution of *P. polysora* isolates. Non‐epidemic region: *P. polysora* is rarely or has not been reported. Epidemic region: *P. polysora* frequently occurred. Overwinter region: *P. polysora* can survive during winter with living maize plants. (B) Principal component analyses plotted by top three PCs. Ellipse encloses isolates from the Philippines that are genetically distant from other isolates. (C) Phylogenetic analysis and population structure of 189 isolates from three tested countries. A total of 6473 highly reliable single‐nucleotide polymorphisms (SNPs) were used to construct the maximum‐likelihood tree. The population structure was analysed by discriminant anaylsis of principal components (DAPC) clustering approach with *K* ranging from 2 to 10. The *K* = 6 was chosen to explain well the population grouping.

RNA‐seq of 193 infected samples yielded an average of 36.2 million reads per sample. Alignment to the dikaryotic genome (GD1913) got 33.58% mean mapping rate (range between 6.81% and 88.90%, Table S1). The aligned reads covered 20,986 out of 40,464 *P. polysora* genes, which were identified by at least 10 samples with more than 5 reads per gene. In total, 73,035 high‐quality single‐nucleotide polymorphisms (SNPs) were identified, of which 53,570 were located within 21,896 genes. The average SNPs per gene is only 2.45, suggesting limited genetic variation across the sampled populations. To enhance the reliability of downstream analyses, we applied stringent quality filters, retaining only SNPs with a missing rate < 50% and a maximum allele count > 4. This resulted in a high‐confidence set of 6473 SNPs, which were distributed across 6108 genes. Among these, 4071 genes (66.7%) contained only one SNP, while the vast majority (6102 genes, 99.9%) harboured fewer than five SNPs. Consistent with our previous findings (Li et al. 2025), these RNA‐seq‐derived SNPs showed strong concordance with genome resequencing data, supporting their robustness. SnpEff annotation classified 62.3% as missense and 34.99% as silent mutations (Table S3). To effectively visualise the differences among samples, we performed principal component analysis (PCA) and found seven Philippine samples as a genetically distinct group (Figure 1B). To definitively exclude potential contamination by common corn rust (*Puccinia sorghi*), we constructed a phylogenetic analysis using two genetic markers: rDNA internal transcribed spacer (ITS) and large subunit of rRNA (LSU). The sequences of *P. polysora* were retrieved from assembled transcripts using Trinity v. 2.9.1 (Haas et al. 2013), while sequences of other rusts were downloaded from NCBI (Table S4, File S1). The phylogenetic reconstruction yielded robust support (bootstrap value = 100) for the classification of all samples as conspecific with *P. polysora* GD1913 (Figure S1), further supporting this conclusion. Read mapping rates revealed an average rate of 16.85% (10.42% to 26.99%, Table S1) to the GD1913 genome across all samples, a rate consistent with other confirmed *P. polysora* specimens. Crucially, mapping rates to the *P. sorghi* genome (Rochi et al. 2018) remained consistently below 1% in all cases. These combined results provide conclusive evidence that all sampled isolates, including the phylogenetically most divergent Philippine clade (TH1), represent the rust species *P. polysora*.

In discriminant analysis of principal components (DAPC) results, the optimal number of clusters was inferred using Bayesian information criterion (BIC) (Figure S2). The highly divergent clade PH1 was distinct, followed by the Chinese endemic clades (CN1 to CN4) when *K* = 3. When *K* = 4, the highly virulent Chinese clade CN3 (previously designated as G6 by Li et al. [2025]) was distinguished. The Thai endemic clade TH1 was recognised at *K* = 5, and the Chinese clade CN4 and the Philippine clade PH2 were further distinguished at *K* = 6. With increasing values of *K*, two clades Mix1 and Mix2, which include samples from three countries, could not be distinctly assigned a single colour, indicating their admixed origins. In addition, the Chinese clade CN2, including samples mostly from southern border regions such as Hainan, Yunnan and Guangxi, exhibited admixture of different clusters. Our analysis indicated that, alongside the two international pandemic clades (Mix1 and Mix2), each country maintained its own distinct cluster, including TH1 in Thailand, CN1 in China and PH1 in the Philippines, demonstrating how regional adaptation coexists with global dispersal in this pathogen system.

### 2.2 Host Selection Shaped Endemic Clade

**FIGURE 2:**
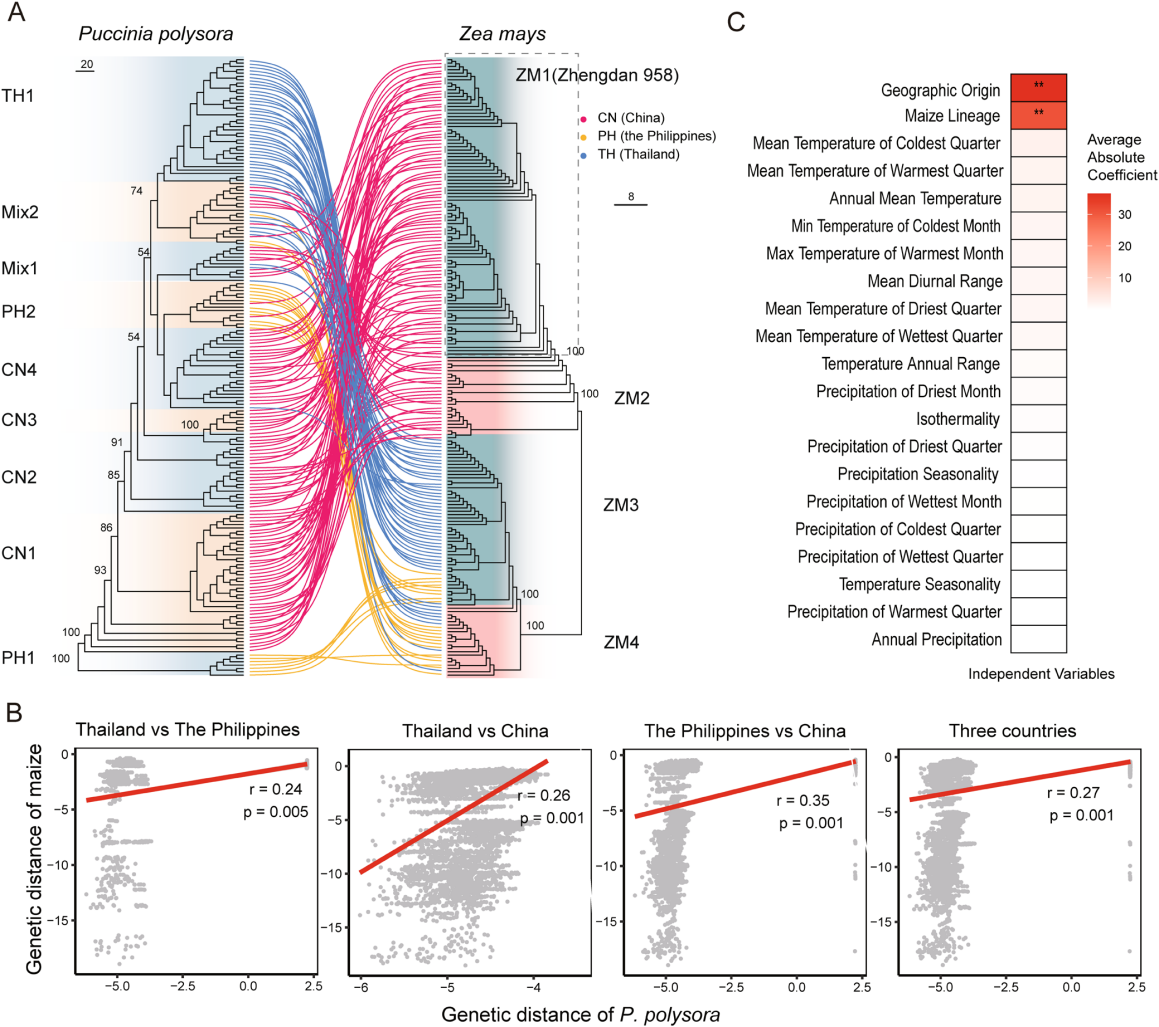
Factors driving lineage differentiation of *Puccinia polysora*. (A) Comparison of phylogenetic trees constructed by single‐nucleotide polymorphisms (SNPs) from *P. polysora* (left) and maize hosts (right). The SNPs used for tree construction were called from the SCR metatranscriptomic data and mapped to the dikaryotic genome of *P. polysora* GD1913 and the maize B73 v5, respectively. The maize tree was constructed using 23,439 SNPs. The same sample was connected by lines between the trees, and line colours represent the geographic origin. The ZM1 lineage in the dashed box includes samples collected from Zhengdan 958. (B) Mantel test of genetic distance between trees constructed by SNPs from host and pathogen. The significant positive correlation (*r* value) in three national pairs and all countries suggested co‐evolution between pathogen and host. (C) Correlations of geographic origin and 19 environmental factors on phylogenetic lineage differentiation. **p < 0.01.

Through comparative phylogenetic analysis of SNPs derived from both *P. polysora* and its maize host, we established clear correlations between their genetic backgrounds (Figure 2A). A total of 23,439 high‐quality SNPs were obtained by mapping metatranscriptomic RNA sequences to the maize reference genome. Most maize varieties from China, Thailand and the Philippines exhibited country‐specific clustering (Figure 2A). ZM1 and ZM2 are from Chinese samples, and ZM1 included the RNA samples derived from Zhengdan 958. Both ZM3 and ZM4 are from tropical regions. Most of ZM3 is from Thailand and ZM4 from the Philippines.

Comparison of the topologies of the two trees revealed that *P. polysora* isolates in the same endemic clade were predominantly from the same clade in the maize varieties tree (Figure 2A). A Mantel test of genetic distances between the two trees revealed significant positive *r* values (0.24–0.35, *p* < 0.01) (Figure 2B), suggesting the co‐evolution of *P. polysora* along with the maize varieties of each country and indicating the important roles of host selection in shaping endemic lineages. Incorporating environmental factors into the analysis revealed that host selection and geographic origin had the highest impact on lineage differentiation (Figure 2C, Tables S8 and S9). This strongly suggests that although gene flow was among the three countries, each country still maintained its own local endemic lineage due to strong host selection.

### 2.3 Virulence Variation

**FIGURE 3:**
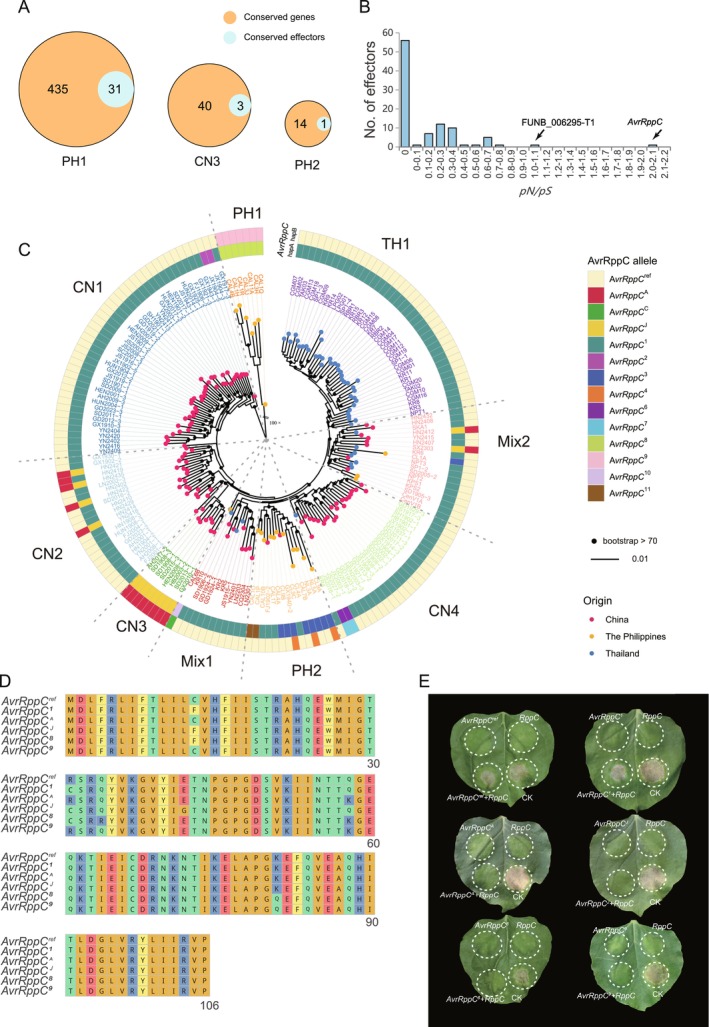
Virulence variation of *Puccinia polysora* in China, Thailand and the Philippines. (A) Number of effectors harbouring clade‐specific variants. Circles represent the number of genes with nonsynonymous mutations in each category. (B) Summary of *pN*/*p* *S* ratio of effectors. (C) Allelic diversity of *AvrRppC* across all studied samples. (D) Amino acid alignments of *AvrRppC* with five newly identified alleles. (E) Coinfiltration assay in *Nicotiana benthamiana*. The coinfiltration of the construct carrying *Avr1‐CO39* and *RGA5* was performed as a positive control (CK); the coinfiltration of the construct carrying the *RppC* genomic DNA construct or the construct with the empty vector were performed as negative controls.

Effectors are important proteins for disease development and represent one of the key virulence factors for plant infection and host–pathogen interactions. To investigate the virulence potential and differentiation among *P. polysora* populations, we characterised the variants in effector genes and identified 1339 SNPs including 219 nonsynonymous variants across 161 effector genes (Figure S3A). Specifically, 37 of them showed lineage specificity over 27 effectors. Three genetic clades (PH1, CN3 and PH2) exhibited unique variants in 32 effector genes. A total of 37 clade‐specific variants were identified (Figure 3A, Figure S3B), with the most clade‐specific variants of effectors discovered in PH1. The CN3 clade, previously identified as a highly virulent Chinese lineage (Li et al. 2025), carried three specific effector variants, including two *AvrRppC* isoforms (FUNA_023436‐T1/FUNB_024415‐T1) and FUNA_005021‐T1. Clade PH2 shared variants with CN3 in FUNA_023436‐T1, while *AvrRppK* was completely conserved across all tested isolates, indicating strong evolutionary constraint.

The *pN/pS* ratios estimation reflects the signature of selection on these effector genes. 57.7% of the effectors were under positive selection (Figure 3B, Table S5, *pN/pS* > 1), suggesting faster virulence regulation and adaptation of *P. polysora* in responding to pressures from different host resistances. *AvrRppC* was under the strongest positive selection (*pN/pS* = 2.07), which aligned with its high genetic diversity in the population. To date, a total of 17 allele types of *AvrRppC* have been detected (Figure 3C, Table S6), including six novel types discovered in this study (*AvrRppC* ^6^ to *AvrRppC* ^11^) (Figure 3D). The isolates in TH1 and CN1 all carried the dominant allele combination *AvrRppC* ^ref^ + *AvrRppC* ^1^, consistent with that in the GD1913 reference genome and known to trigger the *RppC*‐mediated hypersensitive response (HR) (Figure 3E). In contrast, the CN3 lineage possessed the *AvrRppC* ^A^ + *AvrRppC* ^J^ combination that was capable of evading the recognition of *RppC* (Figure 3E). Previous studies suggested that only the CN3 isolates carry *AvrRppC* ^A^ + *AvrRppC* ^J^ alleles (Liang et al. 2023). However, our study showed that several Hainan samples collected in 2024 in CN2 and Mix2 (HN) also carried these highly virulent alleles. Additionally, a new allele combination *AvrRppC* ^8^ + *AvrRppC* ^9^ was detected only in PH1 isolates. Given the risk of introduction of this highly distinct Philippine lineage to China, we tested the immune recognition between *AvrRppC* ^8^ and *AvrRppC* ^9^ alleles with the corresponding *RppC* via coinfiltration assay in *Nicotiana benthamiana*. The results suggested that they can also evade the recognition of *RppC* (Figure 3E).

### 2.4 Gene Flow and Identity‐by‐Descent Analysis

**FIGURE 4:**
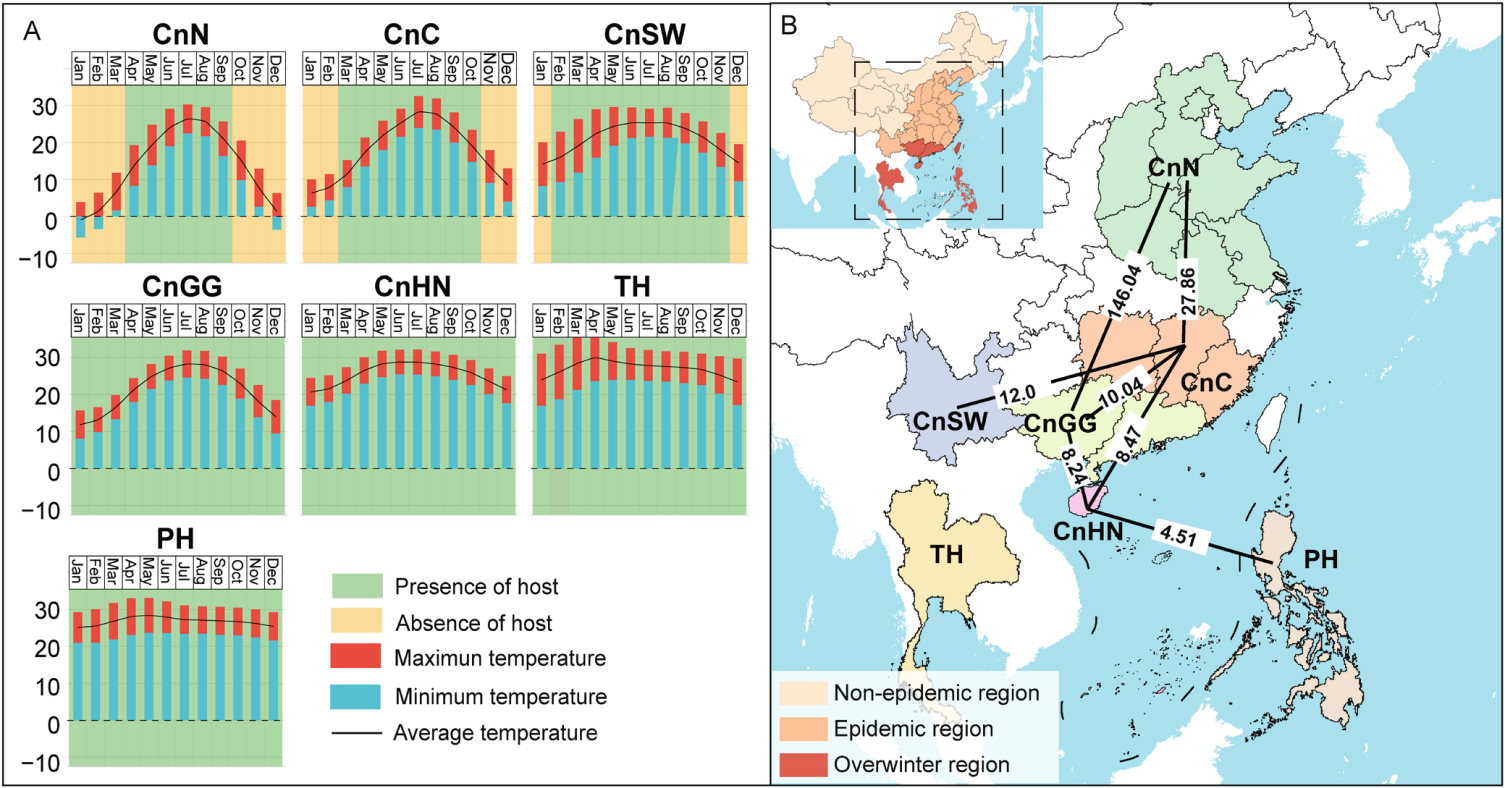
Gene flow and identity‐by‐descent (IBD) tests of seven ecological populations. (A) The maize‐growing period and temperature features of seven ecological regions. The growing periods were recorded following literature (Ekasingh et al. 2004; Gerpacio et al. 2004; Meng et al. 2006). The temperature data were downloaded from https://worldclim.org. CnC, Central China; CnGG, Guangdong and Guangxi, China; CnHN, Hainan, China; CnN, Northern China; CnSW, Southwestern China; PH, Philippines; TH, Thailand. (B) Gene flow between pairs of seven ecological populations. The lines connecting the population pairs indicate that gene flow is not reduced with increased spatial distance (Figure S1). The values on the lines are gene flow calculated by *Nm* = (1 − *F*_ST_)/4*F*_ST_ (Wright 1931).

To estimate the extent and spatial scale of potential gene flow in the studied area, we conducted pairwise identity‐by‐descent (IBD) tests. Samples were divided into seven ecological regions according to host planting period and climate characteristics (Figure 4A). In total, 21 pairwise IBD tests were conducted, and seven pairs showed no significant correlation between genetic distance and geographic distance (*p* > 0.05, Figure 4B, Figure S4). It suggested that the gene flow in these seven pairs was not spatially limited. The potential gene flow was reflected by the lines connecting the seven pairs: PH‐CnHN, CnHN‐CnGG, CnGG‐CnC, CnHN‐CnC, CnSW‐CnC, CnGG‐CnN and CnC‐CnN (Figure 4B). Genetic exchange with PH was detected only in the CnHN region, indicating frequent gene flow between Hainan province of China and the Philippines. In contrast, gene flow between the Philippine populations and other Chinese populations (e.g., CnN and CnC) appeared to be limited. To rule out bias introduced by the highly divergent Philippine population (PH1), we calculated pairwise *F*_ST_ between (i) the Philippine population including PH1 (Phi) and (ii) the Philippine population with PH1 removed (Phi_rm) versus all other populations (Table S7). The results showed that pairwise *F*_ST_ values between Phi and other populations were higher, and some pairs were even higher than 0.25 (a threshold indicating strong differentiation) (Hartl and Clark 2006); however, when PH1 was removed, the *F*_ST_ values decreased to lower than 1 (Table S7), indicating that the pathogens have the potential to overcome spatial barriers and achieve inter‐regional spread. Although a few Thai isolates clustered with Chinese and the Philippine isolates within the pandemic clades, no potential migration was inferred between Thailand and other geographical populations based on the IBD test, indicating that the Thai population may be isolated due to natural barriers to gene flow. IBD analysis showed extensive potential migration among five Chinese five *P. polysora* populations, which is consistent with the low *F*_ST_ values (< 0.03) between population pairs in China (Table S7) with central populations (CnGG and CnC) serving as ecological corridors, the transmission hubs of Chinese *P. polysora*. The absence of gene flow between Hainan (CnHN) and northern populations (CnN) indicates a gradual, stepwise northward spread of the pathogen.

### 2.5 Airflow Trajectory Analysis and Dispersal Network

**FIGURE 5:**
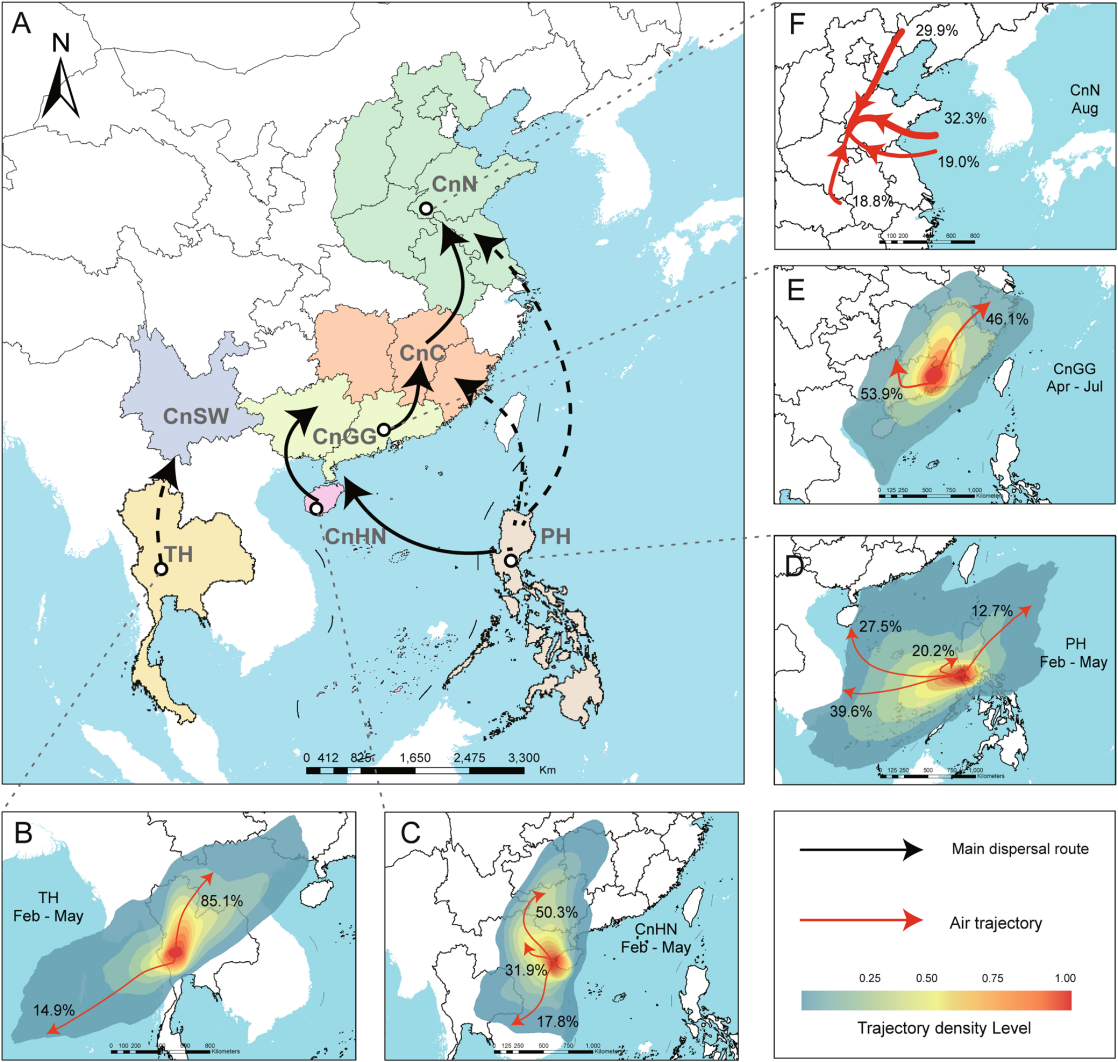
Airflow trajectory analyses. (A) An overview of dispersal network inferred according to air trajectory analyses and gene flow in Figure 4. (B–E) Forward trajectory simulation initiated from representative coordinates in TH, CnHN, PH and CnGG. The red arrows represent clustered air trajectories and the numbers are average trajectory frequency (ATF). (F) Backward trajectory simulation toward to CnN. Trajectories are clustered into four main trajectories and the values represent trajectory frequency.

To determine the migration directions, we performed monthly air trajectory tracking across seven geographical populations to assess potential migration routes (Figure 5A). From November to January, the prevailing airflow of Thailand and the Philippines flows west or southwest (Figure S5), opposite to China's direction, making *P. polysora* dispersal from these countries to mainland China highly improbable. During February–May, SCR's tropical peak season, Thailand's north‐eastward airflows reach Yunnan (YN) and Guangxi (GX), with an average trajectory frequency (ATF) of over 85% (Figure 5B, Figure S6). While Hainan's north‐westward (31.9% ATF) and northward (50.3% ATF) airflows influence CnGG and extend to CnC (Figure 5C, Figure S7).

The Philippine airflows during this period clustered to four major directions, including westward (39.1% ATF) to Vietnam and north‐westward (27.5% ATF) to Hainan and the coastal areas of Guangdong (Figure 5D, Figure S5). After May, although the Philippines airflow continues northward along China's coast, typhoons and flooding reduce maize cultivation, indirectly lowering pathogen inoculum levels in tropical regions, making spore transport to provinces like Fujian unlikely and epidemiologically insignificant (Figure 5D, Figure S5). In contrast, SCR intensively occurs in CnGG during May to July, with airflows primarily directed toward Hunan (53.9% ATF) and central coastal China (46.1% ATF; Figure 5E, Figure S8). By August, when SCR peaks in CnN, external inoculum dominates. Backward trajectory tracking in CnN showed that 18.8% of the airflow carrying inoculum originates from the CnC, while airflow from other directions shows low spore transport potential (Figure 5F, Figure S9).

## 3 Discussion

### 3.1 Host Selection on Endemic Clade

Local adaptation of plant pathogens is mainly driven by the interplay between selection and gene flow (Glais et al. 2014). Rust fungi are capable of long‐distance dispersal that promotes genetic exchange between geographically adjacent areas (Hovmøller et al. 2023). Epidemic lineages often dominate in interconnected areas, as exemplified by the rapid displacement of local populations by the invasive ‘Warrior’ race of *P. striiformis* in Europe (Hovmøller et al. 2016). Our findings indicated the persistent emergence of distinct endemic *P. polysora* clades in China, Thailand and the Philippines, despite pandemic evidence of gene flow (Mix clades in Figure 1C). This paradox can be explained by strong host selective pressure, which plays a crucial role in driving pathogen differentiation. The well‐documented genetic divergence of maize cultivars across geographical regions (Mir et al. 2013) is clearly reflected in our data. For example, the Thai and Philippine tropical maize varieties are separated from Chinese subtropical and temperate varieties, and located in distinct clades, which directly parallels the population structure we observed in their rust pathogens (Figure 2A), highlighting the importance of host–pathogen co‐evolution in shaping local adaptation.

### 3.2 Exploring the SCR Infection Source in China

*Puccinia polysora* faces survival challenges in northern China due to a lack of hosts in winter (Figure 4A) (Meng et al. 2006), yet SCR outbreaks have been reported in the summer maize‐producing area of Huanghuaihai (Liu et al. 2016). While Liu et al. (2018) suggested that the presence of *P. polysora* in Tianjin (northern China) could be attributed to long‐distance wind dispersal from southern China, ISSR analyses (Guo et al. 2013; Yan et al. 2018) found no genetic link between isolates from Hainan and outbreak regions, leaving the origin of northern infections unresolved. Some researchers proposed a hypothesis of foreign origin (e.g., Philippines/Thailand) for Chinese SCR infections (Wang et al. 2020). However, our integrated IBD and trajectory analyses indicated minimal influence from these countries. Instead, strong gene flow connects southern (CnHN/CnGG) and northern China, suggesting that the source of SCR infection in northern China could be primarily of domestic origin. Hainan and Guangdong‐Guangxi may likely serve as *P. polysora*'s invasion bridgeheads in China, following the establishment‐spread model (Bertelsmeier and Keller 2018). In Hainan, the Nanfan Breeding Base, cultivating a diverse range of maize varieties (Yan et al. 2023), may have exerted strong host selection pressure for local adaptation of *P. polysora*.

### 3.3 Global Dispersal History and Current Epidemic Centres of *P. polysora*

**FIGURE 6:**
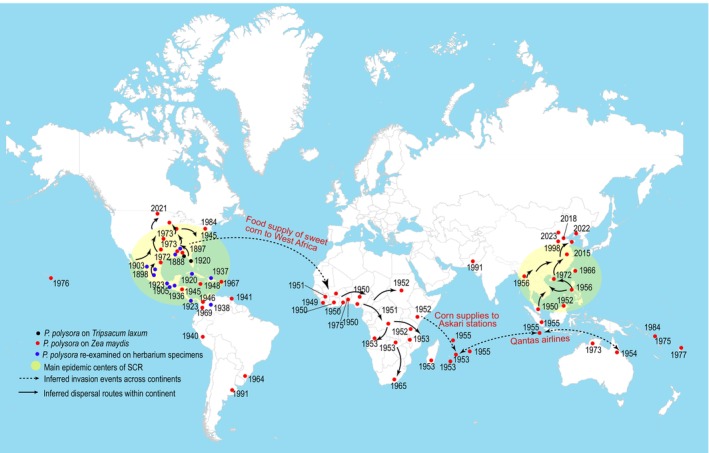
Global invasion history and two current epidemic centers of *Puccinia polysora*. The occurrence map and invasion years are based on data from GBIF.org (accessed 01 April 2025) GBIF Occurrence Download https://doi.org/10.15468/dl.tvq8sn and literature reviews (Cammack 1959; Barker 1969; Duan and He 1984; Agarwal et al. 2001; Liu et al. 2018; Halvorson et al. 2021; Sun et al. 2021). Re‐occurrences of *P. polysora* after 2000 are not labelled.

Understanding and predicting the introduction and dispersal routes of fungal invasions remain challenging (Gladieux et al. 2015). Historical literature review reveals that the global invasion and dispersal routes of *P. polysora* can be traced back to its initial report on *Tripsacum laxum* in Alabama in 1897 (Cammack 1959) (Figure 6). Its occurrence on maize was first documented in Peru in 1940, and subsequent re‐examination of herbarium specimens indicated its presence on maize as early as 1871 (Cummins 1941; Casela and Ferreira 2002). In 1949, outbreaks of SCR in West Africa resulted in severe yield losses (Rhind et al. 1952), probably introduced via US sweet corn imports during and after World War II (Cammack 1959). The disease subsequently spread across the continent, reaching the east coast by 1952 and Mauritius in 1953, and entered the Indian Ocean region (Orian 1954). The global prevalence of SCR had somewhat declined due to the wide application of the *Rpp9* resistance gene against the dominant race PP.9 (Ullstrup 1965; Rodriguez‐Ardon et al. 1980). However, the detection of *Rpp9*‐virulent *P. polysora* in Georgia, United States, in 2008 suggested virulence evolution and adaptation (Dolezal et al. 2009). Recent reports show SCR's expansion into temperate zones; as a result, the United States (Vincelli 2010; Halvorson et al. 2021) and China (Sun et al. 2021) have become the major epidemic centres. In the United States, the extension of the growing season for double‐cropping of maize may facilitate the overwintering and early‐season infection of *P. polysora* (Futrell et al. 1975; Vincelli 2010). The widespread cultivation of susceptible varieties in China has been a key factor in SCR proliferation (Liu et al. 2016). Additionally, climate change, particularly global warming, may also have accelerated the northward expansion of SCR from tropical regions into the temperate zones, making two temperate countries, China and the United States, the current epicentres of SCR outbreaks (Ramirez‐Cabral et al. 2017). Integration of global occurrence data with our surveillance further clarifies SCR's introduction to Asia: a pathogen lineage likely disseminated via wind or human activities underwent clonal propagation, evidenced by genetic homogeneity across most Asian samples (Table S7). Over decades, local adaptation to divergent maize varieties and environmental conditions across tropical, subtropical and temperate zones shaped the extant population structure. The distinct PH1 cluster, exclusively identified in Luzon Island, may represent either a relict of the initial introduction or a novel incursion.

### 3.4 Field Pathogenomics and Virulence Surveillance

The capacity of rust pathogens to adapt and overcome host resistance leads to the rapid emergence of new variants within a few seasons (Möller and Stukenbrock 2017; Saunders et al. 2019), necessitating continuous virulence monitoring for effective disease management (Park et al. 2011). Historical SCR epidemics revealed diverse *P. polysora* races (EA1 to EA3 in Africa, PP.3 to PP.9 in North and Central America, 17 virulence patterns in Brazil, Robert 1962; Ullstrup 1965; Storey and Howland 1967; Casela and Ferreira 2002). However, these races were not directly comparable in terms of virulence, as they were identified using the traditional methods of spore inoculation on maize lines carrying different resistance genes. A recent study employing population genomics identified a highly virulent G6 group carrying the virulent *AvrRppC* allele type (*AvrRppC* ^A^ + *AvrRppC* ^J^) that defeats multiple resistance genes (*RppQ*, *RppD*, *RppC*, *RppP25* and *RppCML496*) (Li et al. 2025), yet its relationship to traditionally defined races remains unclear.

Prior to 2020, the virulent alleles of *AvrRppC* (*AvrRppC* ^A^ + *AvrRppC* ^J^) were only detected in the CN3 (group G6 Li et al. 2025). However, in 2024, six samples collected from Hainan, genetically grouped in CN2 and Mix2, were detected to carry the virulent *AvrRppC* alleles in both nuclei or a single nucleus. This suggested that with the temporal development of SCR in China, the highly virulent *AvrRppC* haplotype is spreading across lineages. Additionally, the selection pressure from the Hainan breeding base may have increased the frequency of highly virulent *AvrRppC* strains, as the base serves as a hub for cultivating a vast array of maize varieties, with numerous maize plants of diverse genetic backgrounds and resistance traits being grown there. Additionally, the highly divergent Philippine clade PH1 carries a distinct *AvrRppC* variation that is capable of overcoming *RppC*‐mediated resistance (Figure 5E). Although currently confined to the Philippines, PH1 poses a potential invasion risk to southern China, particularly Hainan and coastal Guangdong. The high diversity and strong selection pressure of *AvrRppC* in the tested population suggest that rust pathogens can rapidly evolve to adapt to host resistance, necessitating persistent surveillance efforts.

International collaboration to map the global genetic diversity of *P. polysora* is essential for monitoring virulence shifts and population dynamics. Our study represents the first step toward establishing a global surveillance system for *P. polysora*. By employing a field transcriptomic approach, we successfully detected the highly virulent CN3 group (group G6, Li et al. 2025), validating this method, which was originally developed for *P. striiformis* (Hubbard et al. 2015), as a labour‐efficient alternative to traditional spore purification and reproduction. Moreover, with the recent release of the *P. polysora* reference genome (Liang et al. 2023) and the extensive sequence data generated in this study and recent studies (Guo et al. 2024; Liu et al. 2025), omics‐based surveillance strategies are poised for development. For instance, Mobile And Real‐time PLant disEase (MARPLE) diagnostics (MARPLE) targeted resequencing of 1690 polymorphic genes successfully captured the global diversity of *P. striiformis* (Radhakrishnan et al. 2019), while in the case of *P. graminis*, MARPLE targeted to genes related to resistance to the azole and succinate dehydrogenase inhibitor fungicides enables real‐time tracking of potential shifts in fungicide sensitivity (Savva et al. 2025). As the severity of SCR increases, there is an urgent need for a wider collection of *P. polysora* to achieve a better understanding of the diversity, virulence variation and spread of this pathogen for a comprehensive basis of disease control.

## 4 Conclusion

This study employed field transcriptomic data to analyse the dispersal network, virulence variation and regional impacts of *P. polysora* across China, Thailand and the Philippines. As a pioneering effort in global surveillance of maize rust pathogens, our work integrating genetic and atmospheric trajectory analyses revealed limited pathogen migration from Thailand and the Philippines into China. These findings underscore the role of host selection in shaping endemic populations and support a domestic origin for temperate epidemics in China. Therefore, deploying disease‐resistant crop varieties targeting endemic lineages is the current priority for disease control. Notably, the highly divergent *P. polysora* lineage (PH1) in Luzon Island may result from either genetic drift of historical lineages or invasion of contemporary exotic lineages and hints at greater global genetic diversity than previously recognised. This lineage possesses new *AvrRppC* alleles capable of evading *RppC‐*mediated resistance, representing a potential biosecurity threat to neighbouring regions. By reconstructing the historical dispersal patterns and elucidating contemporary transmission networks in China and Southeast Asia, this study provides critical foundations for establishing a global surveillance framework for *P. polysora* and emphasises a comprehensive control strategy integrating internal management and external prevention.

## 5 Experimental Procedures

### 5.1 Sample Collection and Transcriptome Sequencing

A total of 193 *P. polysora*‐infected maize leaves were collected for RNA sequencing, including 116 from China, 50 from Thailand, and 27 from the Philippines (Figure 1A, Table S1). Of these, 27 Chinese samples and all international samples were collected directly from the field and stored in RNA Later stabilisation solution (Invitrogen, Thermo Fisher Scientific) before being sent back for extraction. Other Chinese samples were recovered from purified isolates stored at −80°C, and RNA was extracted from the intensively infected leaves (~5 cm long) of Zhengdan 958 (susceptible variety) in the greenhouse. The inoculation process followed the conditions described in Liang et al. (2023). Macro‐RNA from each sample was extracted using TRIzol reagent (Invitrogen). The quality and quantity of RNA were examined by 4200 Bioanalyzer (Agilent). The ~350 bp library was prepared from each sheared RNA and sequenced on the Illumina NovaSeq 6000 platform, generating 150‐bp paired‐end reads at Annoroad Gene Technology Co. Ltd. (Beijing, China).

### 5.2 Variant Calling

Paired reads were first trimmed using Trimmomatic v. 0.36 (Bolger et al. 2014) with parameters of ‘ILLUMINICLIP 2:30:10 LEADING 3, TRAILING 3, SLIDINGWINDOW 4:10, MINLEN 50’. To extract reads from pathogen and host plant respectively, the clean reads were mapped to the reference genomes of *P. polysora* (GD1913, GCA_025617555.3, Liang et al. 2023) and maize (B73 v5, GCA_902167145.1, Hufford et al. 2021) separately using Hisat2 v. 2.2.1 (Kim et al. 2019) with default settings. For *P. polysora*, the tandem mapping strategy with two haplotypes together as the reference was applied as described by Li et al. (2025). SNPs were called jointly across all samples using Freebayes v. 1.3.2 (Garrison and Marth 2012), and filtered with the parameter ‘QUAL > 20 & QUAL/AO > 10 & SAF > 0 & SAR > 0 & RPR > 1 & RPL > 1 & AC > 0 & FS < 60’ using vcffilter of VCFlib v. 1.0.0 (Garrison et al. 2022). The biallelic SNPs with depth > 5, missing data < 50%, and minor allele count > 4 were retained for following analyses for both *P. polysora* and *Zea mays*. SNP annotation was performed based on genomic locations and predicted coding effects, according to the genome annotation of *P. polysora* GD1913 using SnpEff v. 4.3 (Cingolani et al. 2012).

### 5.3 Population Genetic Analysis

We used the filtered biallelic SNPs to estimate the population structure of *P. polysora*. To effectively visualise the differences among samples, we performed PCA to reduce dimensionality using PLINK v. 1.9 (Purcell et al. 2007) and visualised using ggplot v. 3.50 (Ginestet 2011). The maximum‐likelihood tree of *P. polysora* was constructed using IQ‐tree v. 2.2.0 (Nguyen et al. 2015), of which the best‐fitting model was determined by BIC using the parameter ‐m MFP + ASC.

To reveal the genetic variation within populations, we employed DAPC, a model‐free *K*‐means clustering method that does not rely on the assumption of free recombination, because the *P. polysora* population in the studied area is mainly clonal (Li et al. 2025). The R package adegenet v. 2.0.1 (Jombart 2008) with genetic clusters (*K*) ranging from 2 to 10 was stimulated. The optimal *K* value was determined by the lowest BIC value generated in the find.cluster function. To compare the genetic diversity and variation between subgroups, *π* values were calculated for each subgroup in sliding windows of 1 Mb and the level of genetic differentiation (*F*_ST_) was calculated between subgroups using VCFtools v. 0.1.13 (Danecek et al. 2011).

The effects on population subdivision were estimated on both abiotic (climate data) and biotic (host variety) factors. For the abiotic factor, gridded weather and climate data of the 5 m resolution were downloaded from the WorldClim database v. 2 (https://worldclim.org), and the 19 Bio factors were extracted using the R package Raster v. 3.6 and Geodata v. 0.6. A mixed logistic regression was applied to determine the significance of each factor to population subdivision. For the biotic factor, the host variety was defined according to the maize phylogenetic tree that was constructed using SNPs called against the maize genome B73 v5 (Hufford et al. 2021) as described above. To compare topological congruence between phylogenetic trees constructed by host (maize) and pathogen (*P. polysora*), a tree‐based Mantel test was performed, and the genetic distance matrices of the two trees for each pair of the identified clusters were calculated using the R package vegan v. 2.5.7 (Oksanen et al. 2024).

### 5.4 Virulence Variation and Transient Expression in *N. benthamiana*

The virulence of rust fungi is based on the immune recognition between their secreted effectors and the host's resistance genes (Figueroa et al. 2020). To elucidate the virulence variation in *P. polysora* populations, we detected SNP variants on effectors predicted in the GD1913 genome (Liang et al. 2023), with particular focus on two effectors identified previously: *AvrRppK* (Chen et al. 2022) and *AvrRppC* (Deng et al. 2022). In the Chinese *P. polysora* population, *AvrRppK* is conserved and has broad‐spectrum resistance, whereas *AvrRppC* is highly differentiated with one lineage carrying a unique combination of *AvrRppC* alleles (*AvrRppC* ^A^ + *AvrRppC* ^J^) that can escape the immune recognition of the major resistance genes in China (Deng et al. 2022; Liang et al. 2023). Their allelic diversity across all samples was documented to trace the prevalence of the avirulence genes within international samples. Selective pressure of effectors was measured employing the ratio of *pN/pS* (nonsynonymous to synonymous nucleotide substitution rate ratio) (Schloissnig et al. 2013). The ratio was calculated based on the nonsynonymous substitution rate (*pN*) and the synonymous substitution rate (*pS*), which were determined using the following formulae: *pN* = DN/NN and *pS* = DS/NS, where DN is the number of nonsynonymous substitutions, NN is the number of nonsynonymous sites, DS is the number of synonymous substitutions, and NS refers to the number of synonymous sites. *pN/pS* > 1 indicated positive selection, whereas *pN/pS* < 1 indicated negative selection.

To verify the virulence phenotype of different allele types, we cloned their coding sequences and transformed them into *Agrobacterium tumefaciens* GV3101 via electroporation. Transformants were identified by PCR using primers specific for each construct. The transformed agrobacteria were cultured in Luria Bertani liquid medium, grown overnight at 28°C with shaking at 200 rpm, and harvested by centrifugation at 4000 rpm for 10 min. The bacterial pellet was resuspended in the infiltration buffer (10 mM MES, pH 5.6, 10 mM MgCl_2_ and 150 mM acetosyringone) and adjusted to OD_600_ of 0.8 before infiltration into the leaves of 4‐week‐old *N. benthamiana* plants. Leaves were scored for visible cell death (HR) between 48 and 72 h after infiltration and with the positive control of a recognised *R*–*Avr* pair (*Avr1*‐*CO39* and *RGA5* [Cesari et al. 2013]). Each allele type was tested in three independent experiments, with five replicates per experiment.

### 5.5 IBD Analysis

To assess whether genetic divergence increases with geographic separation, we examined *P. polysora* populations from three counties under Wright's IBD model (Wright 1943; Slatkin 1993). Although IBD analysis does not qualify gene flow directly, the slope of the regression between pairwise *F*_ST_ (GenDis) and geographic distance (GeoDis) provides a robust proxy: a significant positive slope indicates that migration (gene flow) declines with distance, while the intercept reflects baseline differentiation at zero distance (Bohonak 2002; van Strien et al. 2015). Given that the suitable temperature and the availability of living maize plants are essential for the survival of *P. polysora*, we classified all samples into seven ecological populations based on maize planting cycles, climate conditions and geographical locations (Figure 4A): three tropical regions with year‐round maize cultivation (TH, PH and CnHN), one subtropical region with year‐round maize cultivation (CnGG) and three temperate regions with varying maize cultivation gaps (CnSW, CnC and CnN). The GeoDis between each pair of ecological populations was calculated using Euclidean distance based on their coordinates, as implemented in Geosphere v. 1.5–14 (Hijmans et al. 2017). Pairwise GenDis was calculated using the Poppr package v. 2.9.3 (Kamvar et al. 2014). The correlation between GeoDis and GenDis was tested using a Mantel test in R package vegan v. 2.5.7 (Oksanen et al. 2024) with 10,000 permutations. Gene flow (*Nm*) in each pair was calculated using the formula *Nm* = (1 − *F*_ST_)/4*F*_ST_ (Wright 1931).

### 5.6 Airflow Trajectory Analysis

To determine potential dispersal routes of *P. polysora*, we conducted airflow trajectory analyses. The weekly meteorological data during the past 10 years (2015–2024) were downloaded from the National Oceanic and Atmospheric Administration (NOAA) website (ftp://arlftp.arlhq.noaa.gov/pub/archives/gdas1). The trajectory was simulated with the Hybrid Single‐Particle Lagrangian Integrated Trajectory (HYSPLIT) model using the R package splitr (https://github.com/rich‐iannone/splitr) (Draxler 1998; Stein et al. 2015). According to the chronological order of disease occurrence, we selected five representative coordinates from PH, TH, CnHN, CnGG and CnC for forward trajectory simulation and one coordinate from CnN for backward trajectory simulation (Table S2). The simulation periods for each coordinate spanned the whole year, and the trajectory density maps were visualised monthly. The simulation time lasted for 72 h at each coordinate. Both forward and backward trajectories were clustered based on the angle distance clustering method and performed using the R package openair (Carslaw and Ropkins 2012). A total spatial variation method was used to determine the best number of clusters. Trajectory density was calculated and visualised using Spatial Analyst Tools/Line Density in ArcMap v. 10.7.

## Author Contributions

**Yuanjie Li:** investigation, resources, data curation, formal analysis, visualization, writing – original draft; **Wiruda Pootakham:** funding acquisition, resources; **Supawadee Ingsriswang:** funding acquisition; **Fe Dela Cueva:** funding acquisition; **Benjamine William Cordez:** resources; **Yusufjion Gafforov:** funding acquisition; **Jintana Unartngam:** investigation, resources; **Lin Liu:** resources; **Guozhi Bi:** investigation, formal analysis; **Peng Zhao:** formal analysis; **K. M. Tsui Clement:** writing – review and editing; **Junmin Liang:** conceptualization, data curation, formal analysis, visualization, writing – original draft; **Lei Cai:** conceptualization, funding acquisition, writing – review and editing.

## Conflicts of Interest

The authors declare no conflicts of interest.

## Supporting information


**Figure S1:** Multilocus (ITS + LSU) phylogenetic tree of *Puccinia polysora* and other related rust species. Isolates in the highly divergent lineage PH1 are labelled in red.

**Figure S2:** Bayesian information criterion (BIC) values inferring number of optimal clusters. Best *K* value at the valley of the curve was 6.

**Figure S3:** Heatmap of SNP variants of *Puccinia polysora* effectors across all isolates. (A) All synonymous and nonsynonymous variants of groups; rows represent SNPs and columns are isolates; (B) group‐specific nonsynonymous variants. Rows are samples and columns are nonsynonymous variants on corresponding effector.

**Figure S4:** Paired isolation‐by‐distance (IBD) analysis among seven ecological populations. The pairs having non‐significant correlation (*p* > 0.5) have potential migration between populations and are highlighted in red box. CnC, central China, CnSW, southwestern China, CnGG, Guangdong and Guangxi; China, CnHN, Hainan, China; TH, Thailand; PH, the Philippines.

**Figure S5:** Monthly forward airflow trajectory stimulation initiated from the Philippines (PH). Linear density was calculated based on the number of trajectories passing through the unit area.

**Figure S6:** Monthly forward airflow trajectory stimulation initiated from Thailand (TH).

**Figure S7:** Monthly forward airflow trajectory stimulation initiated from Hainan, China (CnHN).

**Figure S8:** Forward airflow trajectory tracking stimulation from Guangdong and Guangxi, China (CnGG).

**Figure S9:** Forward airflow trajectory tracking stimulation from the central of China (CnC).

**File S1:** Sequences of ITS and LSU across all collected samples and other related rust species (.fasta).

**Table S1:** Collecting information and sequencing features of studied samples in this study.

**Table S2:** Representative locations used for trajectory stimulation.

**Table S3:** Functional annotation and distribution of SNPs.

**Table S4:** Reference sequences used for construction of multi‐gene phylogenetic tree.

**Table S5:** The *pN/pS* ratios of effectors containing variants.

**Table S6:** The CDS and amino acid sequences of *AvrRppC* alleles.

**Table S7:** Pairwise *F*st among seven ecological populations.

**Table S8:** Environmental factors for each sample.

**Table S9:** Impact statistics of environmental factors on lineage differentiation.

## Data Availability

The data that support the findings of this study are openly available in National Microbiology Data Center at https://nmdc.cn/resource/genomics/project/detail/NMDC10019241, reference number NMDC10019241.
