# Correction to Biosurveillance of Invasive Southern Corn Rust: Insights Into Recent Migration Patterns and Virulence Variation

**DOI:** 10.1111/mpp.70294

**Published:** 2026-06-05

Li, Y., W. Pootakham, S. Ingsriswang, et al. 2025. “Biosurveillance of Invasive Southern Corn Rust: Insights Into Recent Migration Patterns and Virulence Variation.” *Molecular Plant Pathology* 26, no. 10: e70159. https://doi.org/10.1111/mpp.70159

In the above published article, there were spelling errors in two of the author names:

Wiruda Pootakham should be Wirulda Pootakham.

Yusufjion Gafforov should be Yusufjon Gafforov.

The article has also been updated to reflect the changes.

We apologise for these errors.

