# Supplementary figures and images for: Biosurveillance of Invasive Southern Corn Rust: Insights Into Recent Migration Patterns and Virulence Variation
Source: Mol Plant Pathol. 2025 Sep 30;26(10):e70159. doi: 10.1111/mpp.70159 (PMC12483992; doi:10.1111/mpp.70159)

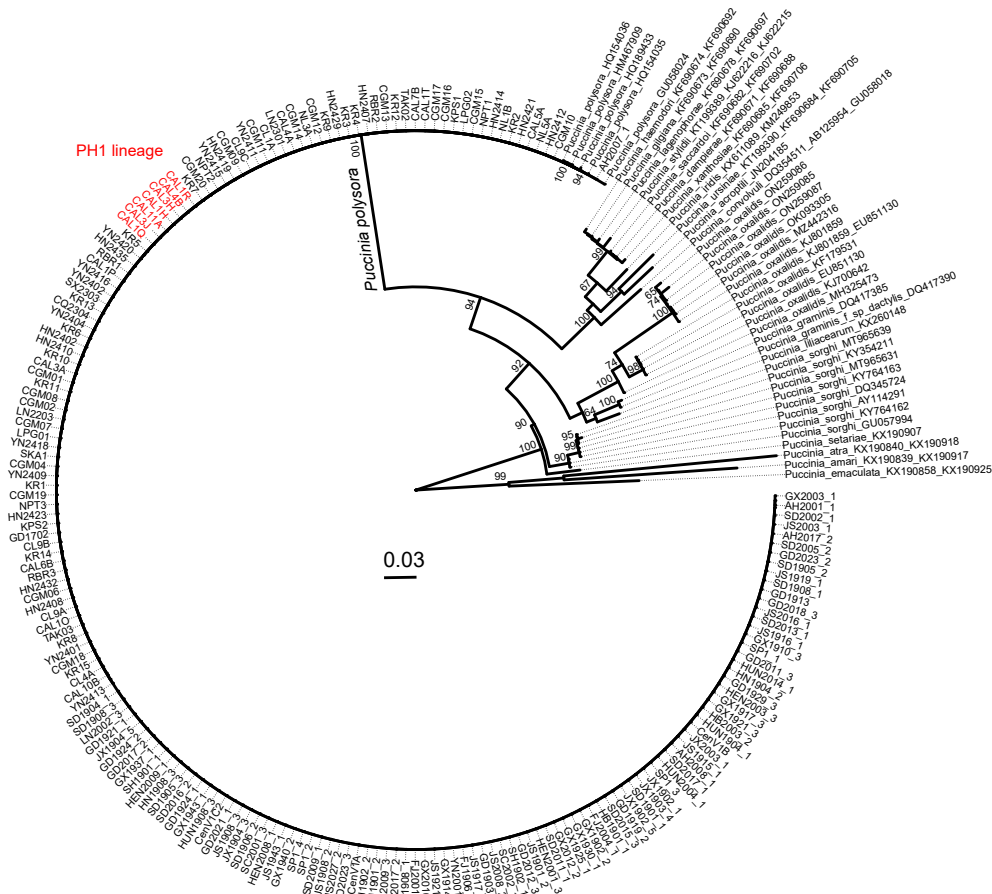

Supplement: Supplementary file 1 — Figure S1: Multilocus (ITS + LSU) phylogenetic tree of Puccinia polysora and other related rust species. Isolates in the highly divergent lineage PH1 are labelled in red. [file MPP-26-e70159-s002.pdf]

**Value of BIC  
versus number of clusters**

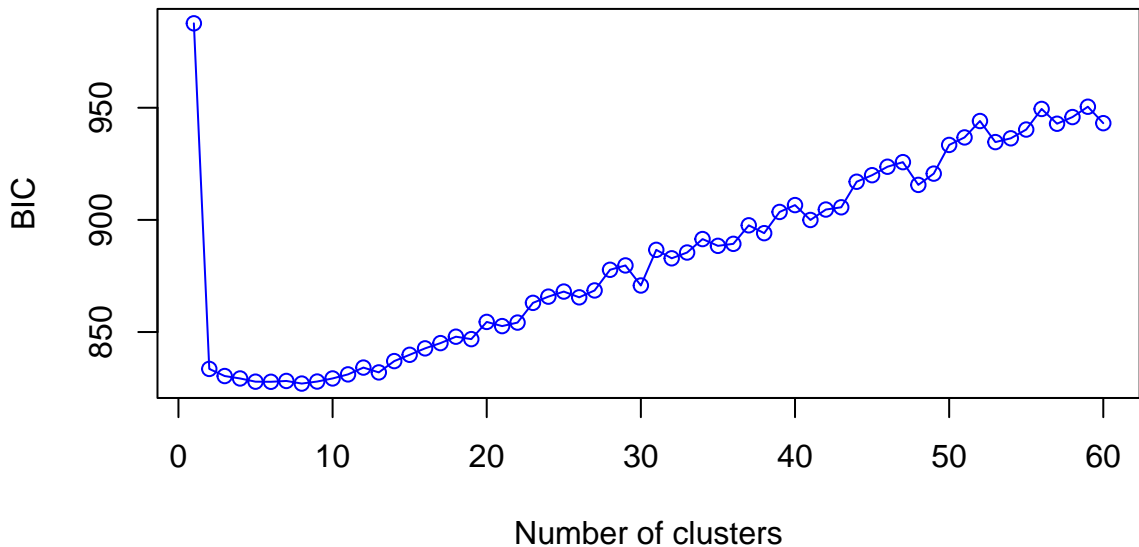

Supplement: Supplementary file 2 — Figure S2: Bayesian information criterion (BIC) values inferring number of optimal clusters. Best K value at the valley of the curve was 6. [file MPP-26-e70159-s003.pdf]

A

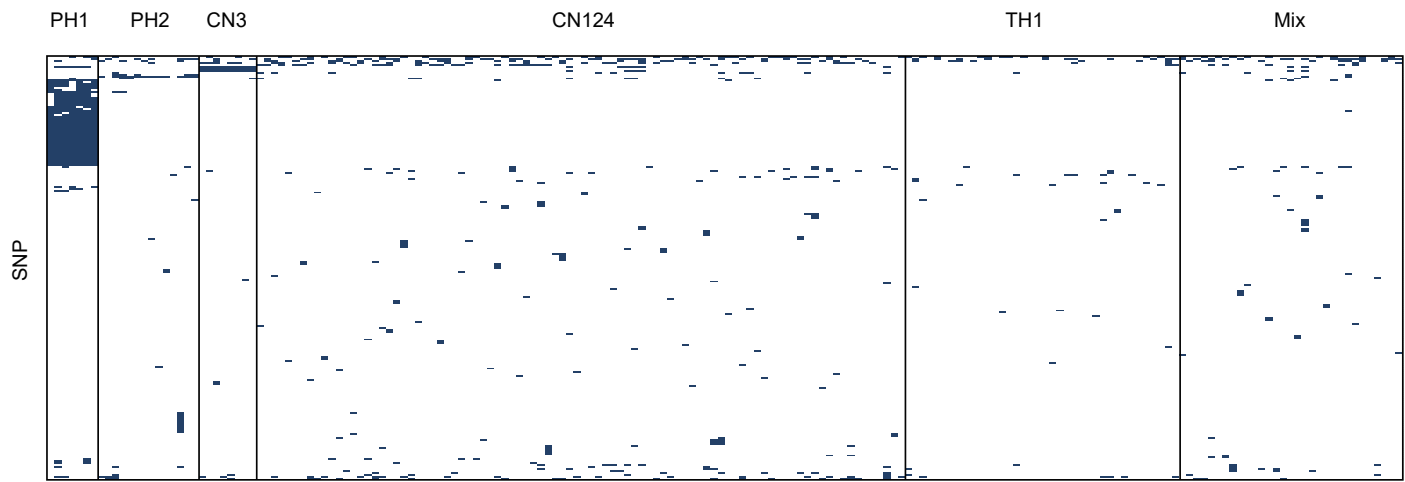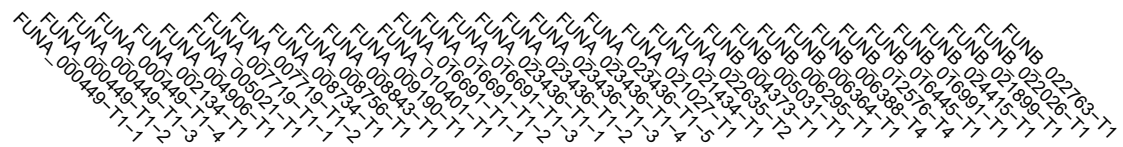

B

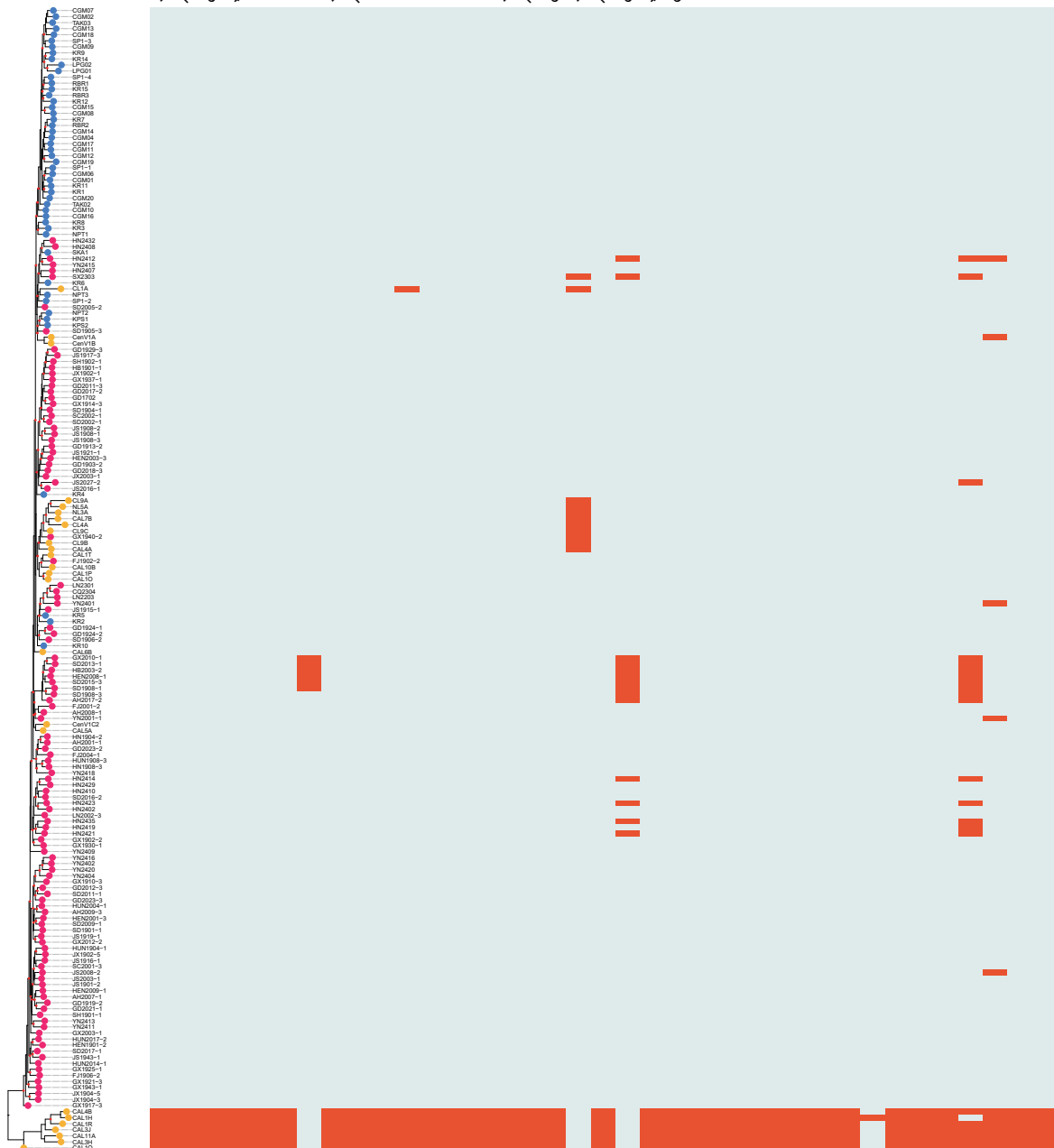

Supplement: Supplementary file 3 — Figure S3: Heatmap of SNP variants of Puccinia polysora effectors across all isolates. (A) All synonymous and nonsynonymous variants of groups; rows represent SNPs and columns are isolates; (B) group‐specific nonsynonymous variants. Rows are samples and columns are nonsynonymous variants on corresponding effector. [file MPP-26-e70159-s009.pdf]

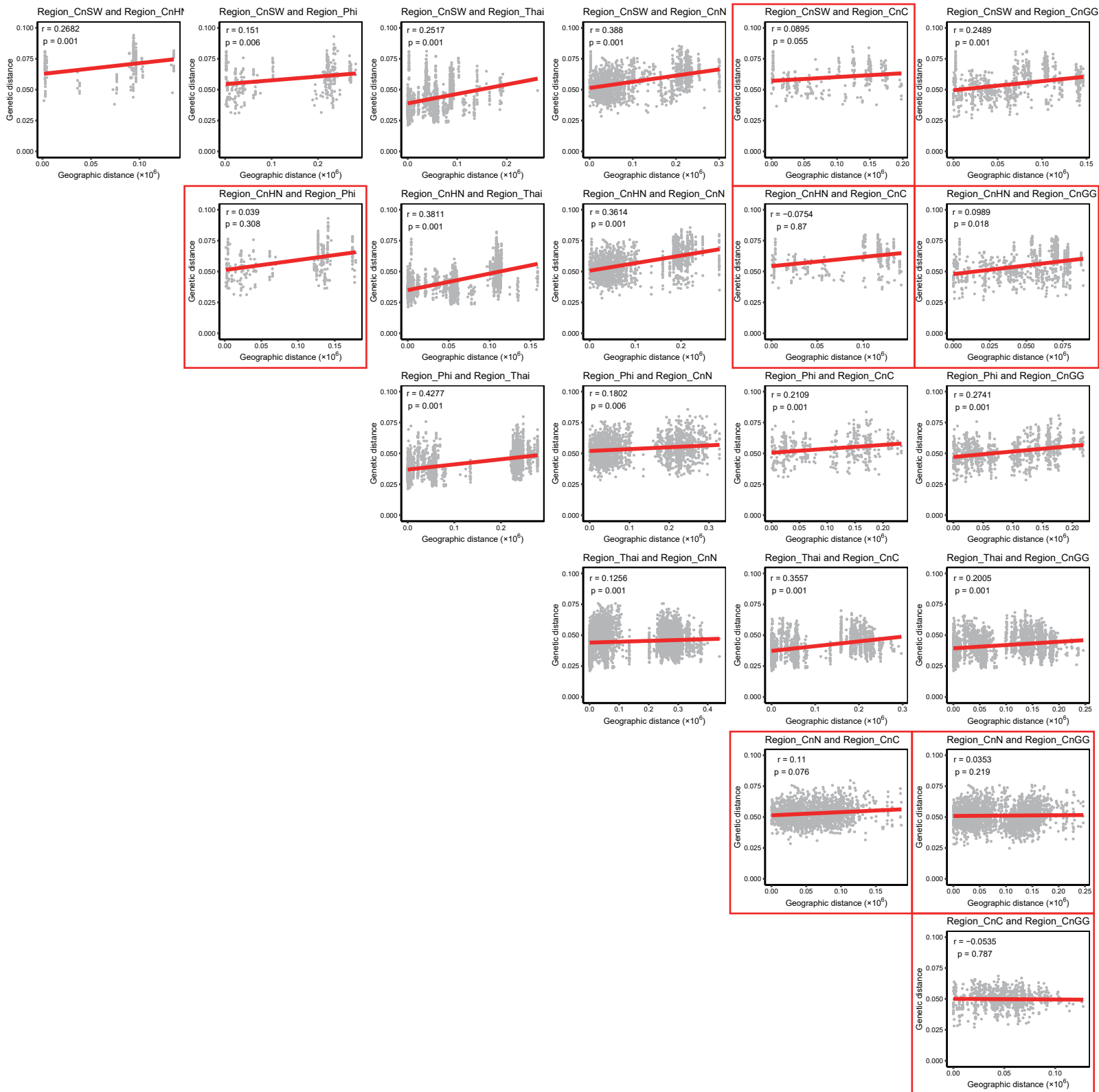

Supplement: Supplementary file 4 — Figure S4: Paired isolation‐by‐distance (IBD) analysis among seven ecological populations. The pairs having non‐significant correlation (p > 0.5) have potential migration between populations and are highlighted in red box. CnC, central China, CnSW, southwestern China, CnGG, Guangdong and Guangxi; China, CnHN, Hainan, China; TH, Thailand; PH, the Philippines. [file MPP-26-e70159-s005.pdf]

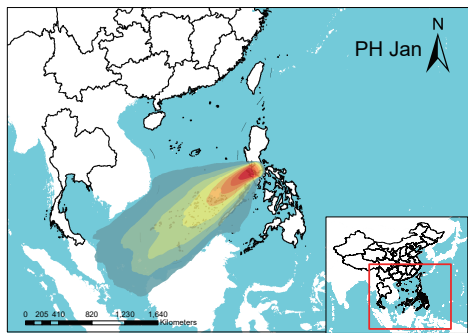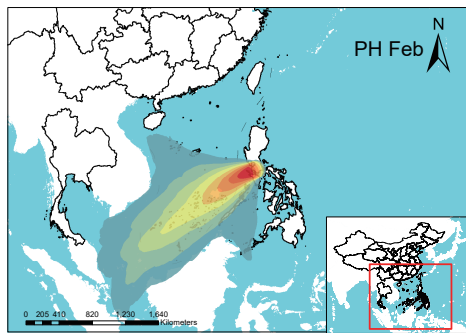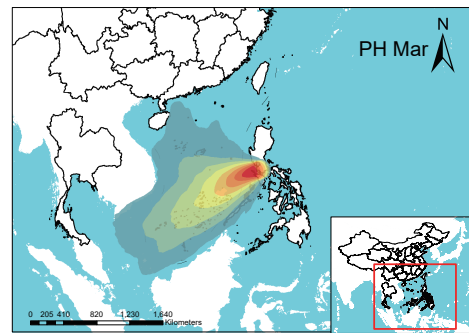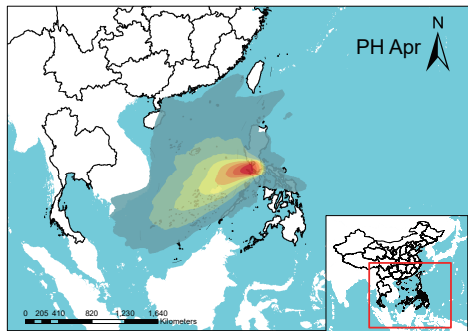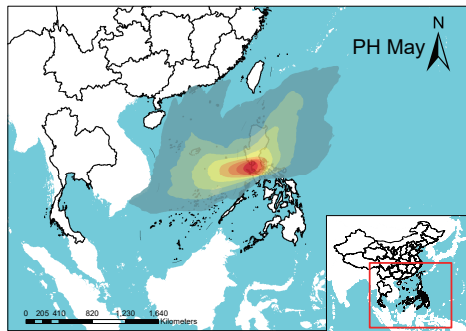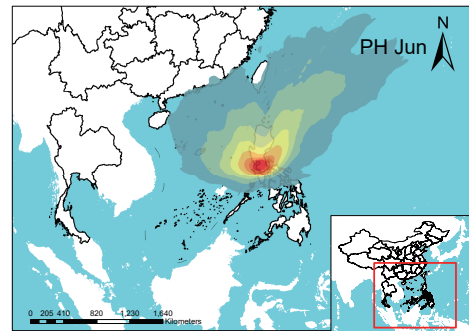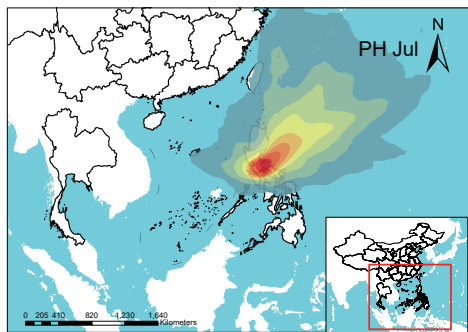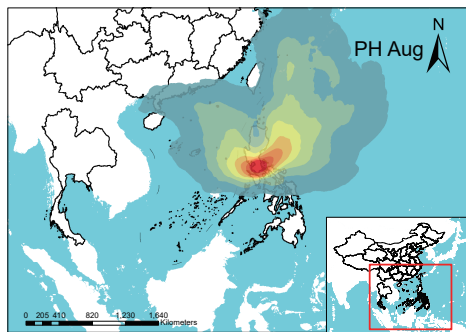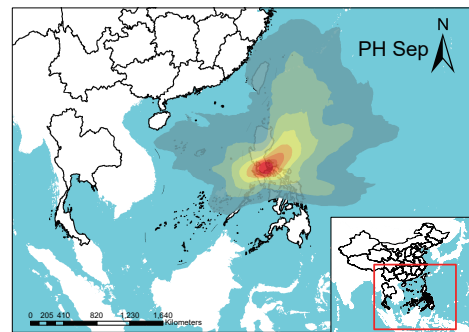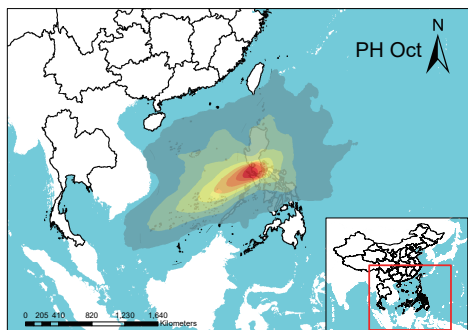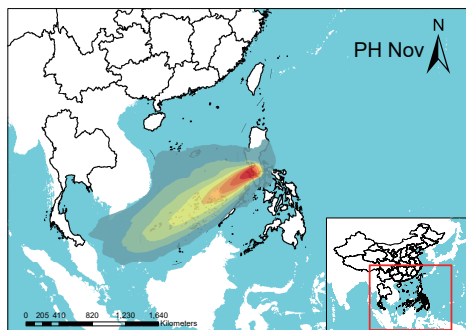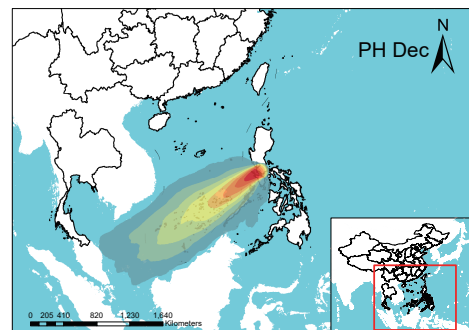

Supplement: Supplementary file 5 — Figure S5: Monthly forward airflow trajectory stimulation initiated from the Philippines (PH). Linear density was calculated based on the number of trajectories passing through the unit area. [file MPP-26-e70159-s017.pdf]

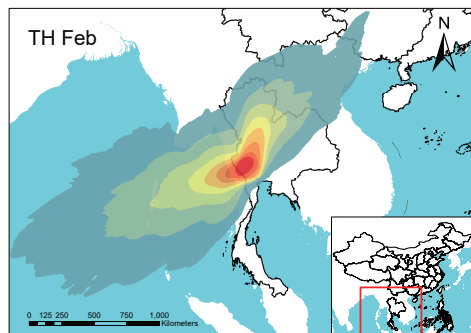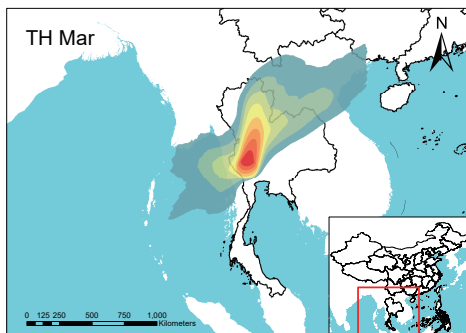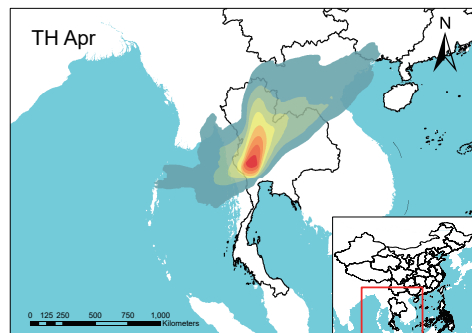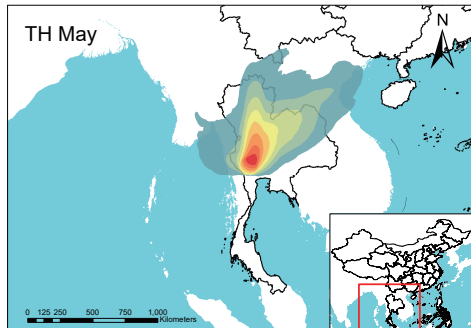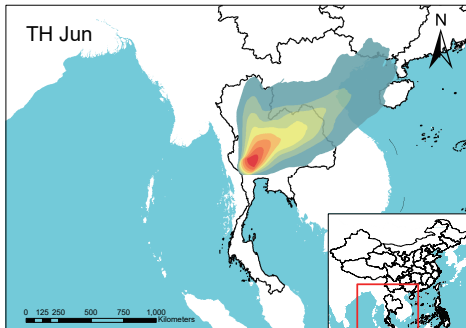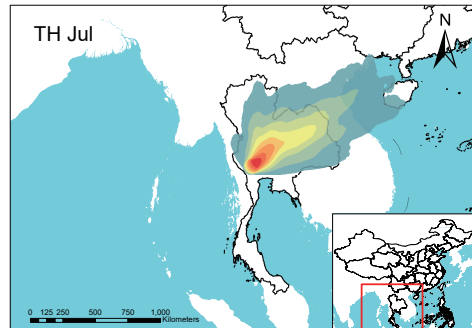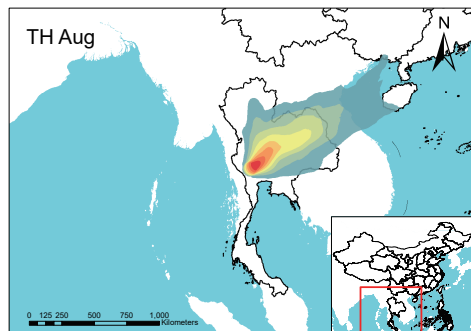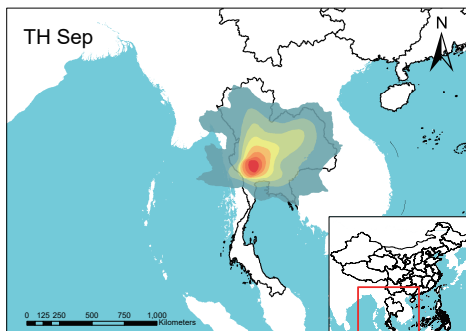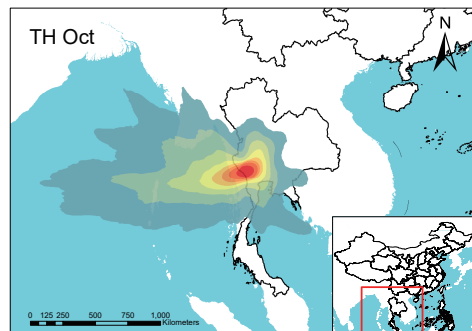

Supplement: Supplementary file 6 — Figure S6: Monthly forward airflow trajectory stimulation initiated from Thailand (TH). [file MPP-26-e70159-s016.pdf]

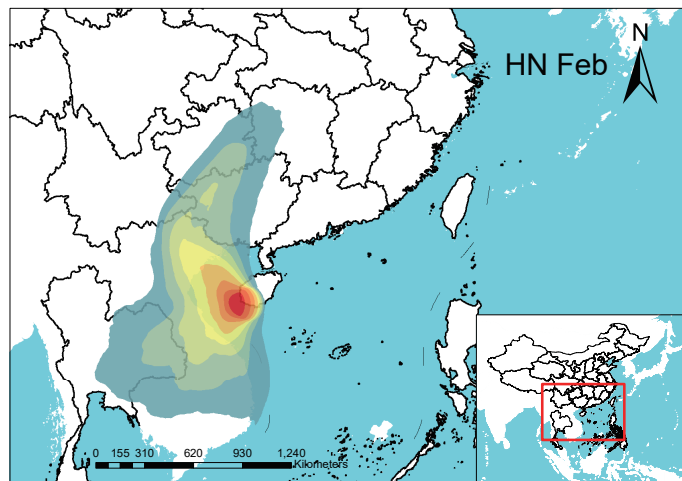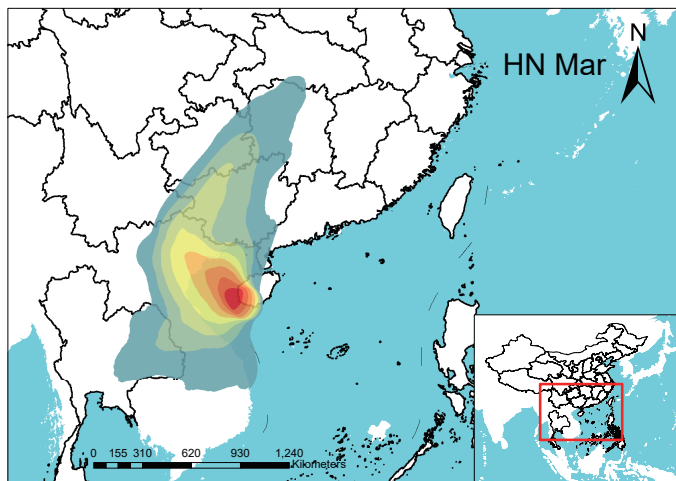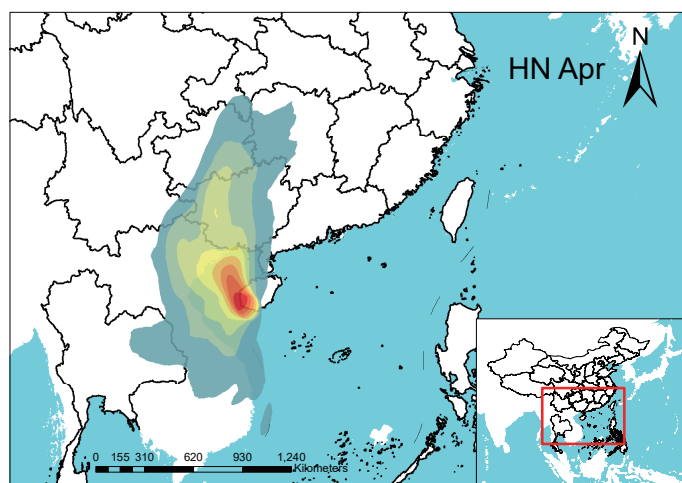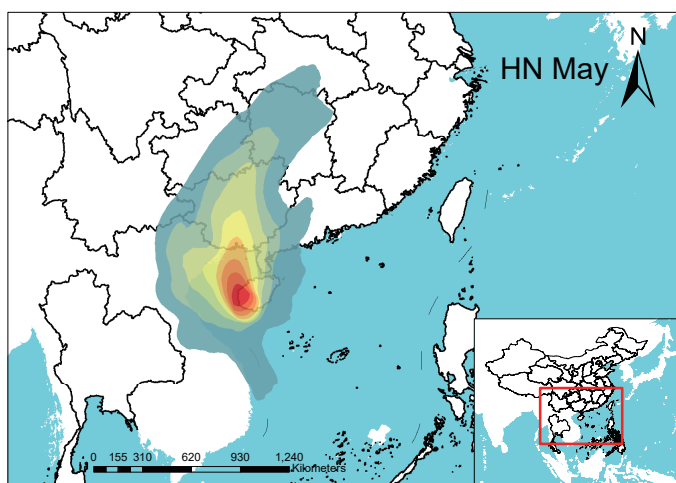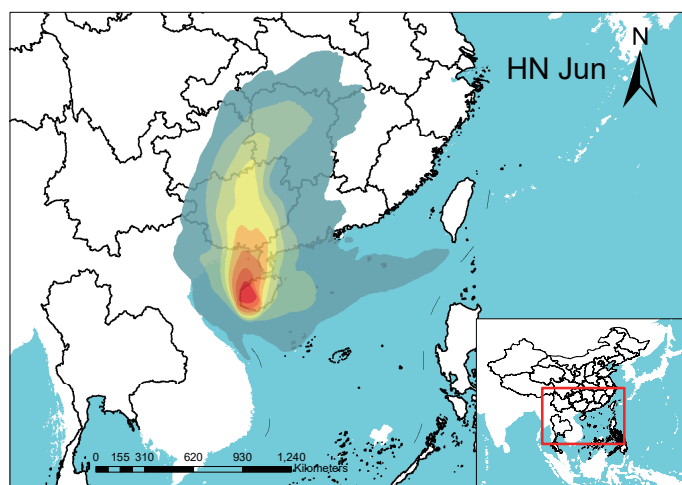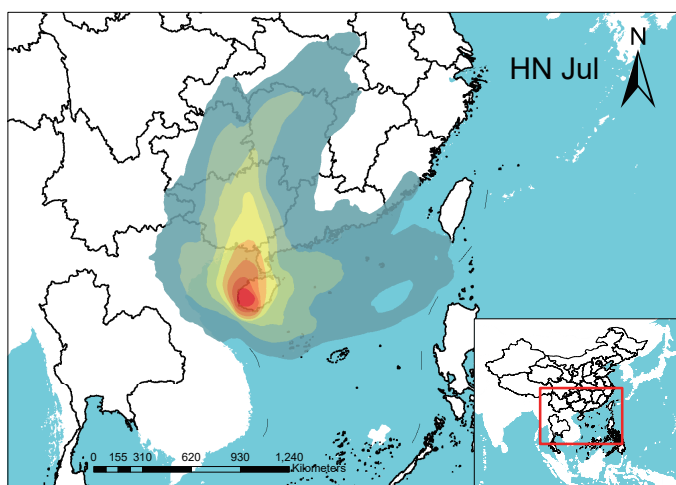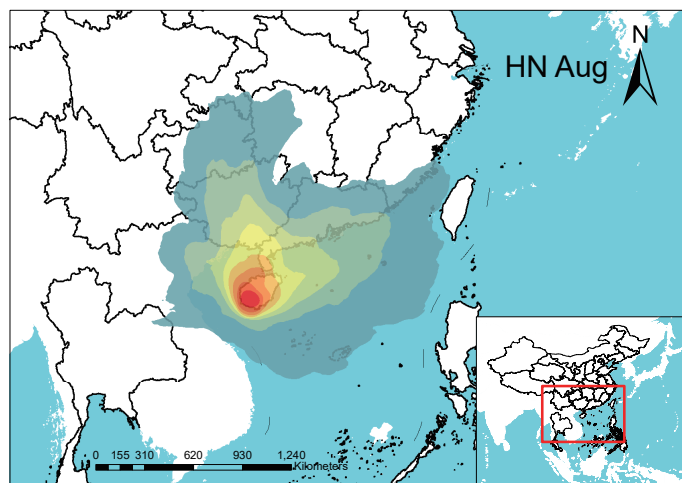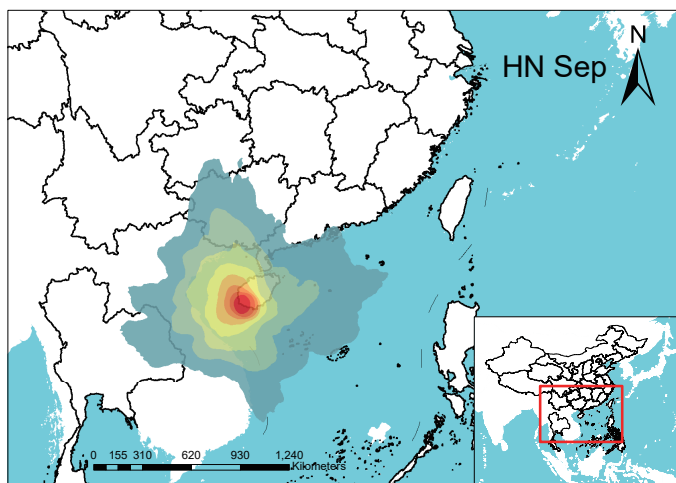

Supplement: Supplementary file 7 — Figure S7: Monthly forward airflow trajectory stimulation initiated from Hainan, China (CnHN). [file MPP-26-e70159-s007.pdf]

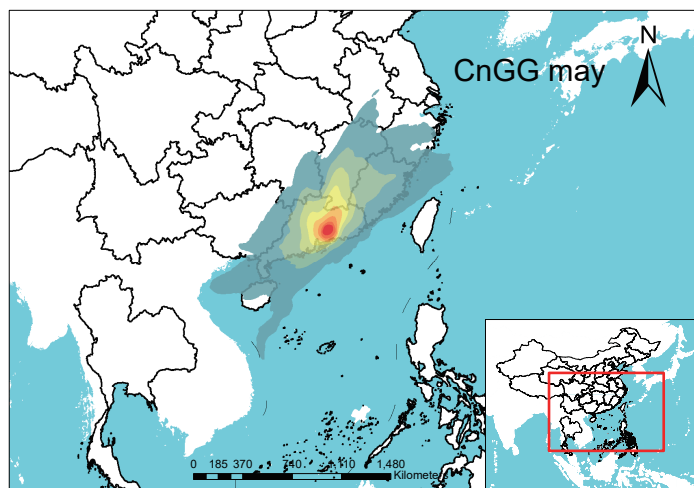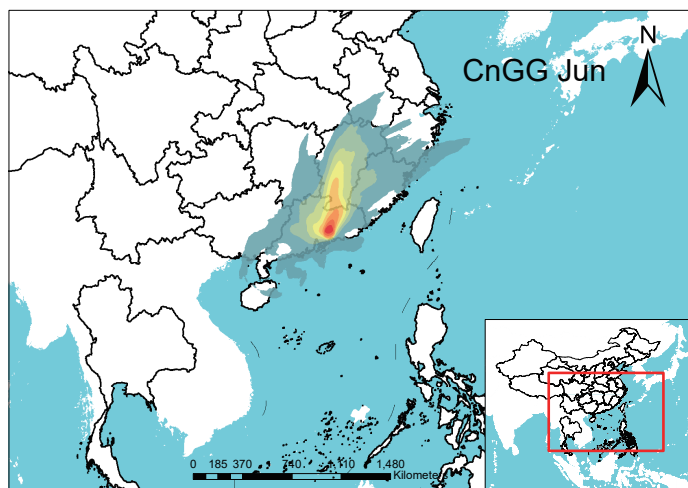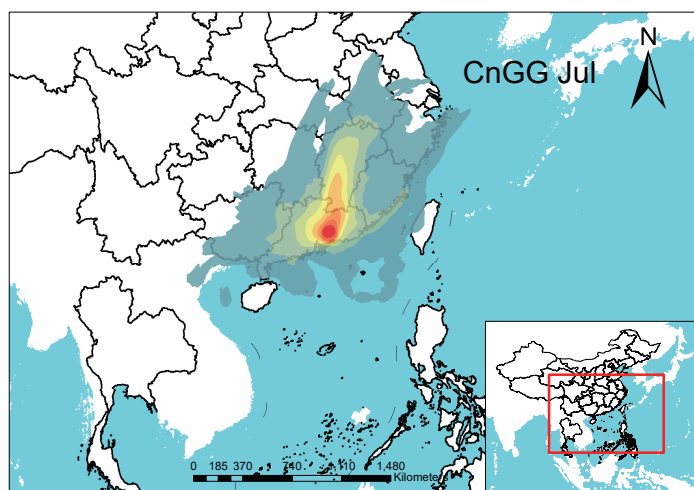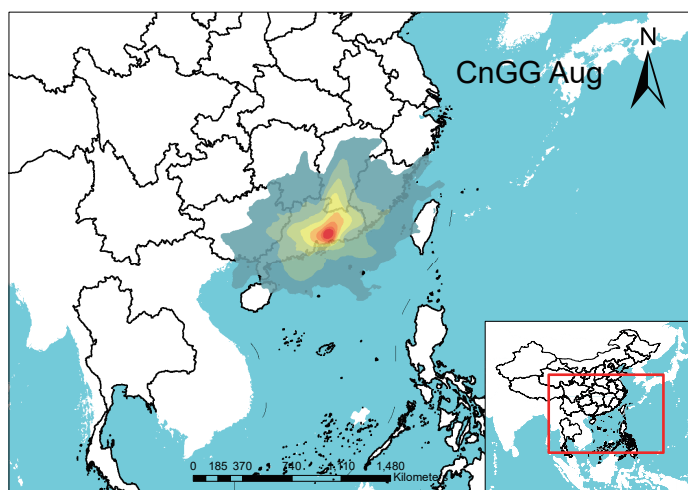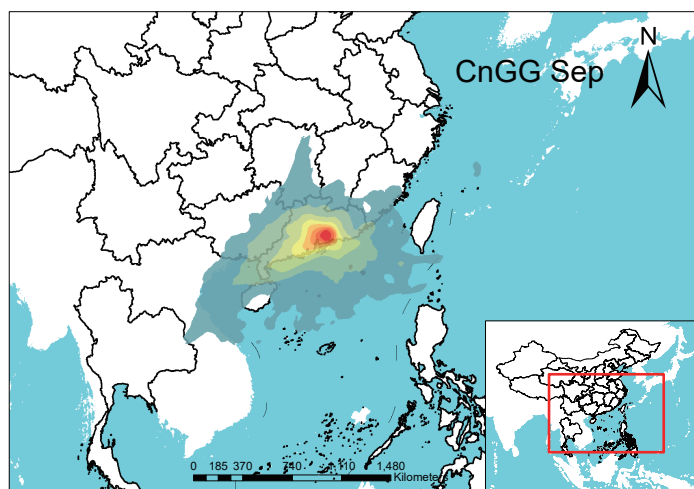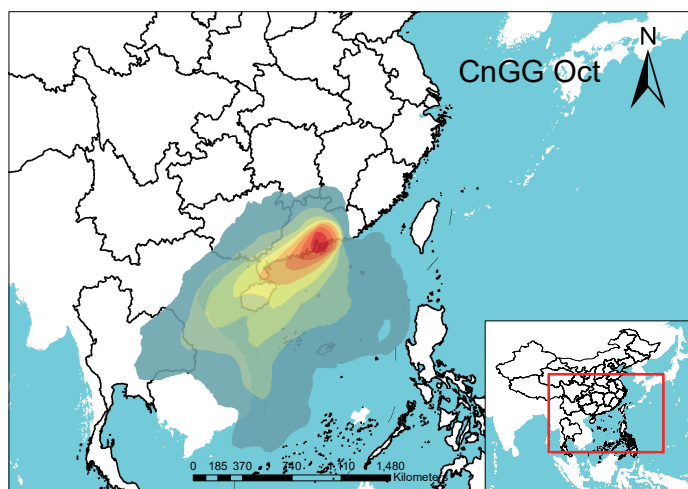

Supplement: Supplementary file 8 — Figure S8: Forward airflow trajectory tracking stimulation from Guangdong and Guangxi, China (CnGG). [file MPP-26-e70159-s012.pdf]

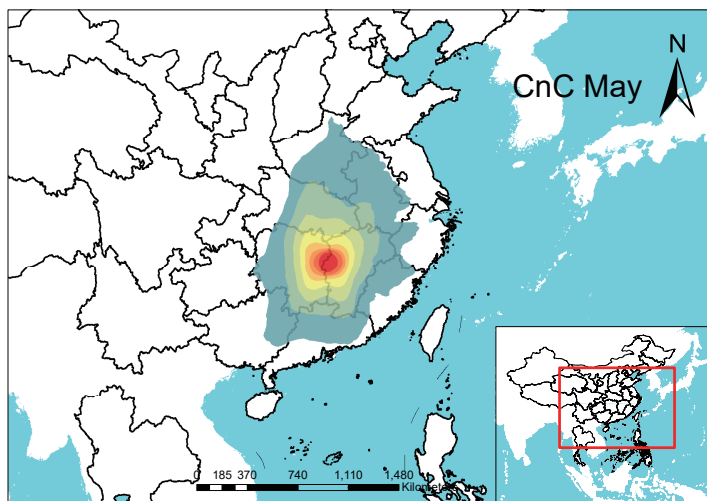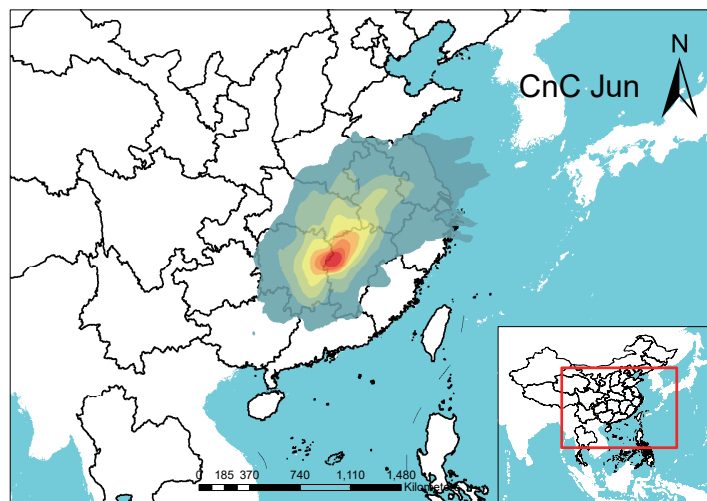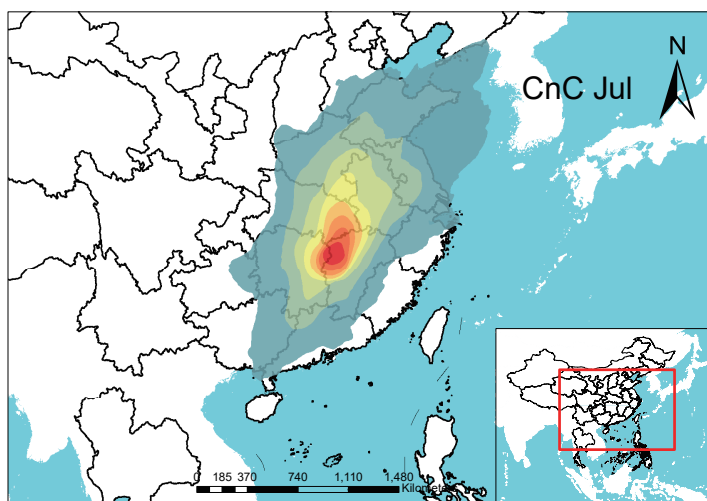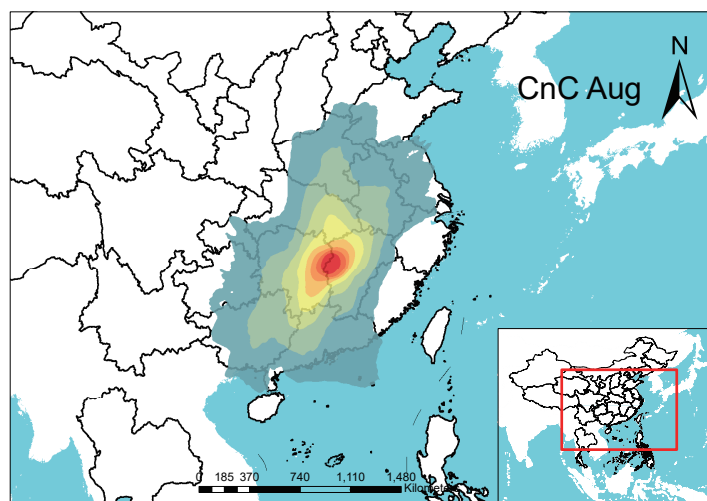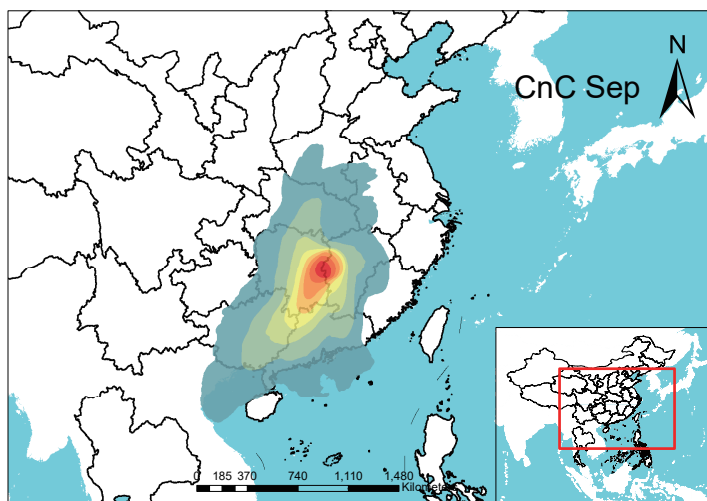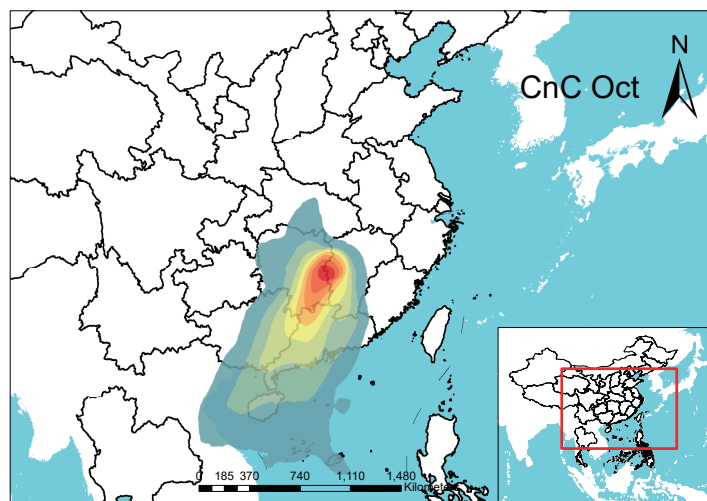

Supplement: Supplementary file 9 — Figure S9: Forward airflow trajectory tracking stimulation from the central of China (CnC). [file MPP-26-e70159-s001.pdf]
